# Qingre Xingyu recipe exerts inhibiting effects on ulcerative colitis development by inhibiting TNFα/NLRP3/Caspase-1/IL-1β pathway and macrophage M1 polarization

**DOI:** 10.1038/s41420-023-01361-w

**Published:** 2023-03-08

**Authors:** Liqin Ning, Ningyuan Ye, Bai Ye, Zhiwei Miao, Tingting Cao, Weimin Lu, Danhua Xu, Chang Tan, Yi Xu, Jing Yan

**Affiliations:** 1 Chinese Medicine Master Studio, The Affiliated Hospital of Nanjing University of Chinese Medicine, Nanjing, 210029 P. R. China; 2 First Clinical Medical College, Nanjing University of Chinese Medicine, Nanjing, 210023 P. R. China; 3 Department of Gastroenterology, The Affiliated Hospital of Nanjing University of Chinese Medicine, Nanjing, 210029 P. R. China; 4 Department of Gastroenterology, Zhangjiagang TCM Hospital Affiliated to Nanjing University of Chinese Medicine, Suzhou, 215600 P. R. China; 5 Department of Internal Medicine, The Affiliated Hospital of Nanjing University of Chinese Medicine, Nanjing, 210029 P. R. China; 6 Key Laboratory for Metabolic Diseases in Chinese Medicine, First Clinical Medical College, Nanjing University of Chinese Medicine, Nanjing, 210023 P. R. China

**Keywords:** Cell biology, Diseases

## Abstract

As a chronic inflammatory bowel disease, ulcerative colitis (UC) imposes a significant burden on public healthcare worldwide due to its increasing morbidity. Chinese medicines are regarded as potent therapeutic agents for UC treatment with minimal side effects. In the present study, we sought to determine the novel role of a traditional medicine Qingre Xingyu (QRXY) recipe in the development of UC and aimed to contribute to the currently available knowledge about UC by exploring the downstream mechanism of QRXY recipe in UC. Mouse models of UC were established by injections with dextran sulphate sodium (DSS), where the expression of tumor necrosis factor-alpha (TNFα), NLR family pyrin domain containing 3 (NLRP3), and interleukin-1β (IL-1β) was determined followed by an analysis of their interactions. The DSS-treated NLRP3 knockout (^−/−^) Caco-2 cell model was successfully constructed. The in vitro and in vivo effects of the QRXY recipe on UC were investigated with the determination of disease activity index (DAI), histopathological scores, transepithelial electrical resistance, FITC-dextran, as well as cell proliferation and apoptosis. In vivo and in vitro experiments indicated that the QRXY recipe reduced the degree of intestinal mucosal injury of UC mice and functional damage of DSS-induced Caco-2 cells by inhibition of the TNFα/NLRP3/caspase-1/IL-1β pathway and M1 polarization of macrophages, and TNFα overexpression or NLRP3 knockdown could counterweigh the therapeutic effects of QRXY recipe. To conclude, our study elicited that QRXY inhibited the expression of TNFα and inactivated the NLRP3/Caspase-1/IL-1β pathway, thereby alleviating intestinal mucosal injury and relieving UC in mice.

## Introduction

Ulcerative colitis (UC) is a chronic, relapsing, and remitting disease of the colon and rectum with characteristic inflammatory mucosa ulceration [1]. Several factors have been identified in the occurrence of UC such as genetics, environmental and luminal factors, as well as a mucosal immune disorder [2]. The increasing incidence of UC across the globe, especially in developing countries is primarily attributed to the aging population and prior history of UC diagnoses [3]. The conventional modalities for UC treatment include the administration of mesalamine, corticosteroids, and immunosuppressants [4]. However, the development of new alternatives for the treatment of UC is warranted because of the remission-induced loss of response to first- or second-line therapies and the adverse effects of current therapies [5].

Currently, accumulating evidence has elicited the potential of traditional Chinese medicines as potent therapeutic agents for UC treatment due to their low recurrence rate and few side effects [6]. For instance, Sanhuang Shu’ai decoction has been identified as an alleviating agent for dextran sulphate sodium (DSS)-induced UC by inhibiting inflammation and oxidative stress [7]. Also, the Qingchang suppository and its components have unraveled UC by anti-inflammation through the JAK2/STAT3 pathway [8]. The current study focused on determining the significance of the Qingre Xingyu (QRXY) recipe in the development of UC.

After network pharmacology and in silico analysis, the tumor necrosis factor-alpha (TNFα) (the potential target for QRXY recipe) and NLR family pyrin domain containing 3 (NLRP3) eliciting associations with UC were chosen as study subjects. TNFα is a vital component for the regulation of the gastrointestinal tract in inflammatory bowel diseases including Crohn’s disease or UC [9]. Interestingly, TNFα knockdown was validated as an effective and safe therapy for patients with refractory UC where conventional treatments were deemed ineffective [10]. Moreover, the serum levels of NLRP3 are positively correlated with a serum of interleukin-1β (IL-1β), TNFα, and thereby the severity of UC patients [11]. IL-1β is a highly specific inflammatory cytokine, which was upregulated in active UC [12]. Caspase-1 is an evolutionarily conserved enzyme mediated by inflammation which can cleave and activate several inflammatory cytokines such as IL-1β and IL-18 [13]. Additionally, another study identified the inactivation of the NLRP3/Caspase-1 could facilitate the alleviation of magnesium lithospermate B for acute and chronic colitis [14].

Hence, in light of the aforementioned literature, it was speculated that the inhibition of TNFα/NLRP3/IL-1β/Caspase-1 pathway was a potential therapy for UC, but whether QRXY could exert a repressing role on this pathway in UC along with their specific interaction requires further investigation.

## Results

### QRXY recipe relieves UC in mice by inhibiting the TNFα pathway

**Fig. 1:**
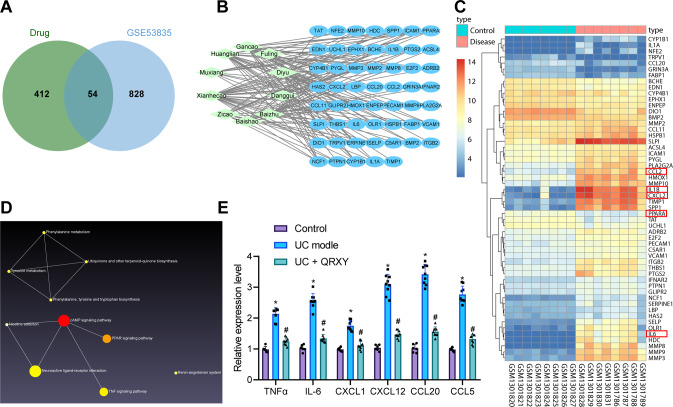
Screening of potential targets and signal pathways for QRXY recipe treatment for UC mice. **A** Venn diagram of the intersection of QRXY potential targets and DEGs in UC-related microarray dataset GSE53835. **B** Regulatory factor network diagram of the main effective components of QRXY. Green represents the main active ingredients of QRXY, while blue represents the main active ingredients of QRXY. **C** Expression heat map of 54 candidate genes in UC-related microarray dataset GSE53835 (color scale blue to red indicates the expression value from low to high). **D** KEGG enrichment network of downregulated genes. **E** The mRNA expression of TNFα pathway-related factors in mouse colon tissues after different treatments determined by RT-qPCR. *n* = 6 in the control group, and *n* = 9 in other groups. *p < 0.05 compared with that of the control, #*p* < 0.05 compared with that of the UC mice. The cell experiments were conducted three times independently.

A total of 466 potential targets of the 10 herbal medicines in the QRXY recipe were identified from the SymMap database. Also, 882 differentially expressed genes (DEGs) were identified after further analysis of gene expression in the UC-related microarray dataset GSE53835. Next, an intersection of potential targets of QRXY and DEGs in UC was chosen, and 54 potential regulatory factors were identified (Fig. 1A, B). The heat map of these 54 candidate genes was plotted by the “pheatmap” package of R language, and the expression of inflammatory factor IL-1β, IL-6, chemokine CXC chemokine ligand-2 (CXCL2), and peroxisome proliferator-activated receptor-α (PPARA) were significantly increased in the UC-related microarray dataset GSE53835 (Fig. 1C).

In order to further predict the pathways involving the 54 candidate factors, the KEGG pathway enrichment analysis of the upregulated genes was conducted using the NetworkAnalyst tool (Fig. 1D; Supplementary Fig. 1), which demonstrated that the predominant concentration of the upregulated genes in the TNFα pathway (*p* = 6.65E−10), while downregulated genes were primarily concentrated in the cMAP pathway (*p* = 0.00372). Our results elicited that inhibition of the TNFα pathway exerts protective activity in UC [15].

To identify whether the QRXY recipe was implicated in the TNFα pathway in UC, the mouse models with UC were established by DSS induction. Reverse transcription-quantitative polymerase chain reaction (RT-qPCR) results showed that the expression of several TNFα pathway-related factors (TNFα, IL-6, CXCL1, CXCL12, CCL20, and CCL5) was increased notably in the colon tissues of UC mice, and was inhibited after QRXY treatment (Fig. 1E).

Accordingly, the QRXY recipe treatment alleviated UC in mice by inhibition of the TNFα pathway.

### TNFα overexpression reverses the therapeutic effect of the QRXY recipe on intestinal mucosal injury in UC mice

**Fig. 2:**
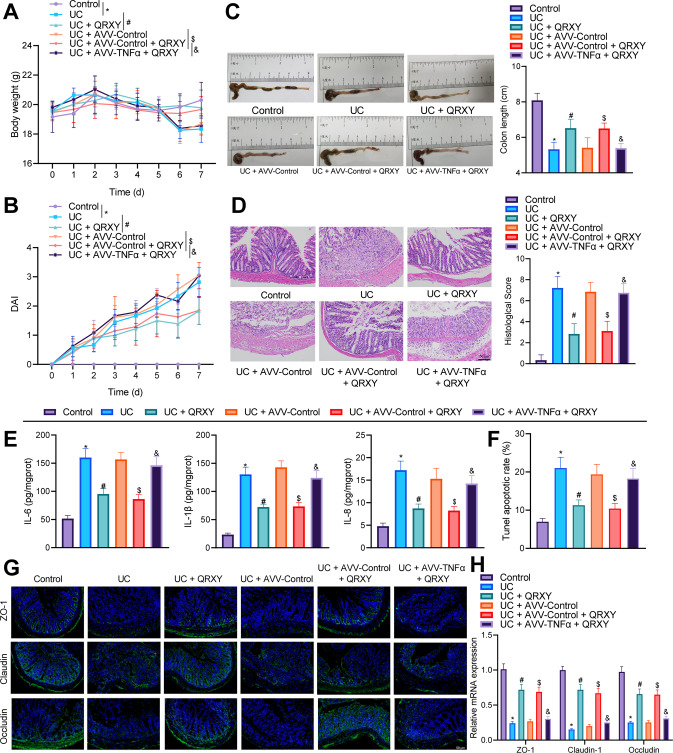
QRXY recipe affects DSS-induced UC in mice through TNFα. **A** Daily body weight change of mice after different treatment protocols. **B** Daily DAI score of mice after different treatment protocols. **C** Quantitative statistics of colon length on the 7th day after DSS treatment protocols. **D** Colon histopathological scores in mice after different treatment protocols. **E** The levels of IL-6, IL-1β, and IL-8 in the colon tissues of mice after different treatment protocols were determined by ELISA. **F** Apoptosis of intestinal mucosal epithelial cells in the colon tissues of mice after different treatment protocols determined by TUNEL assay. **G** The protein expression of ZO-1, Claudin-1, and Occludin in the colon tissues of mice after different treatment protocols determined by immunofluorescence staining. **H** The mRNA levels of ZO-1, Claudin-1, and Occludin in the colon tissues of mice after different treatment protocols were determined by RT-qPCR. *n* = 6 in the control group, and *n* = 9 in other groups. *p < 0.05 compared with that of the control, # *p* < 0.05 compared with that of the UC mice, $*p* < 0.05 compared with that of the UC mice treated by AVV-Control, &*p* < 0.05 compared with that of the UC mice treated by AVV-Control + QRXY. The cell experiments were conducted three times independently.

To explore whether the QRXY recipe alleviated UC in mice through the TNFα pathway, TNFα was overexpressed in UC mice. The body weight of UC mice decreased continuously from the 4th day after DSS treatment compared to control mice and decreased at a slower rate in UC mice after QRXY recipe treatment, but TNFα overexpression reversed the alleviating effect of QRXY recipe on the body weight of UC mice (Fig. 2A). Moreover, the disease activity index (DAI) of UC mice was determined, which was notably higher relative to the control mice, and further decreased after QRXY recipe treatment, but TNFα overexpression resulted in conflicting findings in reference to the effect of QRXY recipe treatment on the DAI of UC mice (Fig. 2B).

The colon length after DSS treatment decreased in UC mice compared to control untreated mice. The colon length increased after QRXY recipe treatment, while TNFα overexpression shortened the colon length (Fig. 2C). As demonstrated by hematoxylin & eosin (HE) staining, incomplete intestinal mucosa, multifocal ulcers, increased inflammatory cell infiltrations, and histopathological scores were evident in UC mice compared to the control mice, while the intestinal mucosal structure was relatively intact, the concentration of inflammatory cells was notably lower, and histopathological scores were reduced after QRXY recipe treatment, while TNFα overexpression exacerbated the disintegration of intestinal mucosal structure (Fig. 2D).

Enzyme-linked immunosorbent assay (ELISA) results manifested that IL-6, IL-1β, and IL-8 expression was notably elevated in the colon tissues from UC mice compared to the control mice, and decreased after QRXY recipe treatment, while TNFα overexpression annulled the inhibition of IL-6, IL-1β, and IL-8 by QRXY recipe treatment (Fig. 2E). Terminal deoxynucleotidyl transferase-mediated dUTP-biotin nick end labeling (TUNEL) results presented that compared to the control mice, the cell apoptosis was notably increased in UC mice, and was reduced with QRXY recipe treatment. Simultaneously, TNFα overexpression promoted cell apoptosis (Fig. 2F). The results of immunofluorescence staining and RT-qPCR showed that the expression of tight junction protein 1 (ZO-1), Claudin-1, and Occludin in the colon tissues of UC mice was reduced compared to the control mice, and increased after QRXY recipe treatment, while TNFα overexpression reversed the promoting effect of QRXY treatment (Fig. 2G, H).

Together, QRXY could reduce intestinal mucosal damage in UC mice, and TNFα overexpression annulled the therapeutic effect of QRXY recipe treatment.

### The therapeutic effect of QRXY recipe on UC in mice by inhibiting TNFα/NLRP3/Caspase-1/IL-1β signal axis

**Fig. 3:**
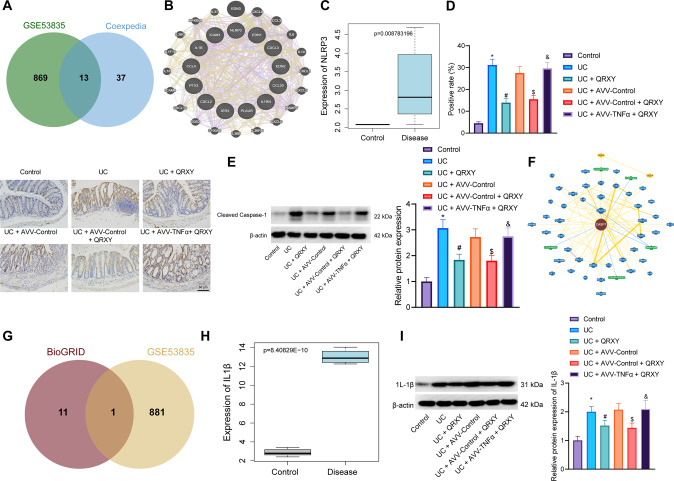
The therapeutic effect of QRXY recipe on UC in mice via regulating TNFα/NLRP3/Caspase-1/IL-1βaxis. **A** Venn diagram of the intersection between TNFα interaction genes and DEGs in UC-related microarray dataset GSE53835. **B** Co-expression relationship network of candidate genes in GeneMANIA database. **C** The expression of NLRP3 in UC. **D** The expression of NLRP3 in the colon tissues of mice after different treatments was determined by immunohistochemistry staining. **E** The expression of cleaved caspase-1 in the colon tissues of mice after different treatment protocols determined by Western blot analysis. *n* = 6 in the control group, and *n* = 9 in other groups. **F** The interaction gene network diagram of Caspase-1 (Alias: CASP1) predicted by BioGRID database. **G** Venn diagram of the intersection between Caspase-1 interaction genes and DEGs in UC-related microarray dataset GSE53835. **H** The expression of IL-1β in UC. **I** The IL-1β expression in the colon tissues of mice after different treatment protocols determined by Western blot analysis. *n* = 6 in the control group, and *n* = 9 in other groups. *p < 0.05 compared with that of the control, #*p* < 0.05 compared with that of the UC mice, $*p* < 0.05 compared with that of the UC mice treated by AVV-Control, &*p* < 0.05 compared with that of the UC mice treated by AVV-Control + QRXY. The cell experiments were conducted three times independently.

To further accurately predict the downstream regulators of the TNFα pathway, the interaction genes of TNF were predicted using the Coexpedia database, and an intersection was chosen between the top 50 genes in the score and the DEGs in the UC-related microarray dataset GSE53835, and 13 candidate genes (CXCL3, PTX3, IL1B, CXCL2, IL1RN, ICAM1, PLAUR, IER3, EDN1, EDN2, CCL4, CCL20, and NLRP3) were finally identified (Fig. 3A).

Next, the gene coexpression relationship network was determined using the GeneMANIA database (Fig. 3B). According to the Gene Score provided by the website, NLRP3 presented the highest score (Supplementary Table 1). On the basis of the differential analysis results of the UC-related microarray dataset GSE53835, the high NLRP3 expression was noted in UC (Fig. 3C). The immunohistochemistry and Western blot analysis demonstrated that NLRP3 and cleaved caspase-1 expression in UC mice was elevated compared to the control mice, and further declined after QRXY recipe treatment, while TNFα overexpression nullified the inhibitory function of QRXY recipe treatment (Fig. 3D, E).

The downstream genes of Caspase-1 were further investigated, the interaction genes of Caspase-1 were predicted using the BioGRID database (Fig. 3F), and an intersection between the gene with Evidence ≥ 2 and DEGs of UC-related microarray dataset GSE53835 was chosen, from which IL1B (Alias: IL-1β) was identified (Fig. 3G). IL-1β presented with notably high expression in the microarray dataset GSE53835 (Fig. 3H). In light of the aforementioned literature, the TNFα/NLRP3/Caspase-1/IL-1βaxis was implicated in the occurrence and development of UC. The results of Western blot analysis determined an elevated expression of IL-1β in the UC mice compared to the control mice, and QRXY recipe treatment reduced the expression of IL-1β in UC mice; however, TNFα overexpression could abrogate the limiting effect of QRXY recipe treatment on IL-1β expression (Fig. 3I).

To conclude, QRXY recipe treatment could serve as a therapeutic factor for UC in mice by inhibition of the TNFα/NLRP3/Caspase-1/IL-1β axis.

### QRXY recipe attenuates the functional damage of Caco-2 cells induced by DSS by inhibiting the TNFα/NLRP3/Caspase-1/IL-1β axis

**Fig. 4:**
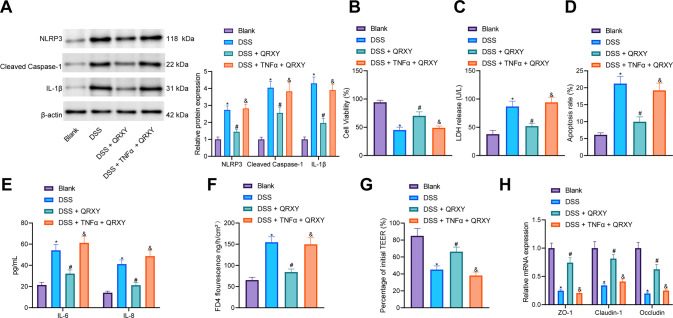
QRXY recipe decreases the functional damage of Caco-2 cells induced by DSS via regulating the TNFα/NLRP3/Caspase-1/IL-1β axis. **A** The expression of NLRP3, cleaved caspase-1, and IL-1β proteins in Caco-2 cells after different treatment protocols determined by Western blot analysis. **B** The viability of Caco-2 cells after different treatment protocols detected by CCK8 assay. **C** The content of LDH in the supernatant of Caco-2 cells after different treatment protocols. **D** The apoptosis of Caco-2 cells after different treatment protocols determined by flow cytometry. **E** The expression of IL-6 and IL-8 in the supernatant of Caco-2 cells after different treatment protocols was detected by ELISA. **F** Determination of FITC-dextran permeability in Caco-2 cells after different treatment protocols. **G** Determination of TEER in Caco-2 cells after different treatment protocols. **H** The mRNA expression of intestinal epithelial barrier markers (ZO-1, Claudin-1, and Occludin) determined by RT-qPCR. The experiment was conducted three times independently. *p < 0.05 compared with that of the blank control, ^#^*p* < 0.05 compared with that of cells treated by DSS, ^&^*p* < 0.05 compared with that of the DSS-treated cells with QRXY treatment.

Next, the mechanism of the QRXY recipe in the treatment of UC mice was further explored through analysis of the TNFα/NLRP3/Caspase-1/IL-1βaxis in Caco-2 cells. As reflected by Western blot analysis, NLRP3, cleaved caspase-1 and IL-1β expression in Caco-2 cells after DSS treatment was increased, while QRXY recipe treatment decreased the aforementioned expression, and TNFα treatment counteracted the inhibitory effect of QRXY recipe on NLRP3, cleaved caspase-1 and IL-1β expression (Fig. 4A). Moreover, cell counting kit 8 (CCK-8) and lactate dehydrogenase (LDH) assays revealed that the cell viability decreased while the content of LDH, cell apoptosis, and the levels of IL-6 and IL-8 were increased in Caco-2 cells after DSS induction treatment, while QRXY recipe treatment enhanced the viability of Caco-2 cells and decreased the content of LDH, cell apoptosis, the levels of IL-6 and IL-8, while TNFα treatment could reverse the effect of QRXY recipe treatment on Caco-2 cells (Fig. 4B–E).

The measurement results of Caco-2 cell fluorescein isothiocyanate (FITC)-dextran permeability and transepithelial electrical resistance (TEER) demonstrated that after DSS induction treatment, TEER decreased and FITC-dextran permeability increased in Caco-2 cells, thus indicative of improved cell permeability. However, QRXY recipe treatment decreased TEER and increased FITC-dextran permeability in DSS-treated Caco-2 cells, leading to weakened cell permeability. Additionally, TNFα treatment could neutralize the effect of QRXY recipe treatment on the permeability of Caco-2 cells (Fig. 4F, G). The RT-qPCR results revealed that the expression of ZO-1, Claudin-1, and Occludin in Caco-2 cells was notably reduced after DSS induction treatment, and QRXY recipe treatment could augment the expression of ZO-1, Claudin-1, and Occludin in DSS-treated Caco-2 cells. Simultaneously, TNFα treatment could also negate the effects of QRXY recipe treatment on the expression of ZO-1, Claudin-1, and Occludin (Fig. 4H).

Therefore, QRXY recipe treatment reduced the functional damage of Caco-2 cells induced by DSS from the repression of the TNFα/NLRP3/Caspase-1/IL-1β axis.

### NLRP3 knockdown blocks the protective effect of the QRXY recipe on DSS-induced Caco-2 cell damage

**Fig. 5:**
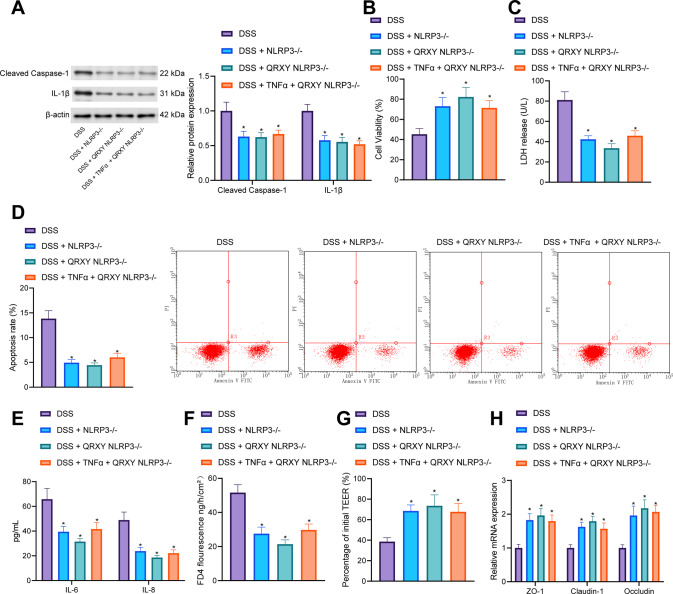
The effect of NLRP3 knockdown on QRXY recipe in attenuating DSS-induced functional damage of Caco-2 cells. **A** The expression of cleaved caspase-1 and IL-1β in Caco-2 cells after different treatment protocols determined by Western blot analysis. **B** The viability of Caco-2 cells after different treatment protocols determined by CCK8. **C** The content of LDH in the supernatant of Caco-2 cells after different treatment protocols. **D** Apoptosis of Caco-2 cells after different treatments determined by flow cytometry. **E** The contents of IL-6 and IL-8 in the supernatant of Caco-2 cells after different treatment protocols were determined by ELISA. **F** The measurement of FITC-dextran permeability of Caco-2 cells after different treatment protocols. **G** The measurement of TEER of Caco-2 cells after different treatment protocols. **H** Detection of intestinal epithelial barrier markers (ZO-1, Claudin-1, and Occludin) of Caco-2 cells after different treatment protocols determined by RT-qPCR. The cell experiment was conducted three times independently. *p < 0.05 compared with that of cells treated by Blank NLRP3^−/−^.

We further explored whether NLRP3 knockdown could influence the therapeutic effect of QRXY on DSS-induced functional damage of Caco-2 cells. As revealed by Western blot analysis, the expression of cleaved caspase-1 and IL-1βwas decreased in DSS-treated NLRP3 knockout (^−/−^) Caco-2 cells in contrast to DSS-induced Caco-2 cells, while no significant difference was identified in NLRP3^−/−^ cells, QRXY + NLRP3^−/−^ cells, or TNFα + QRXY + NLRP3^−/−^ cells after DSS treatment (Fig. 5A). Additionally, CCK-8 and LDH assays demonstrated that the cell viability was enhanced and LDH content was lowered in DSS-treated NLRP3^−/−^ Caco-2 cells in comparison with DSS-induced Caco-2 cells, while no significant difference was noted in the NLRP3^−/−^ cells, QRXY + NLRP3^−/−^ cells, or TNFα + QRXY + NLRP3^−/−^ cells after DSS treatment (Fig. 5B, C).

Flow cytometry and ELISA demonstrated that compared with DSS-induced Caco-2 cells, the cell apoptosis was restricted, accompanied by reduced levels of IL-6 and IL-8 in DSS-treated NLRP3^−/−^ Caco-2 cells, while no significant difference was identified among the NLRP3^−/−^ cells, QRXY + NLRP3^−/−^ cells, or TNFα + QRXY + NLRP3^−/−^ cells after DSS treatment (Fig. 5D, E).

Our results revealed that compared to DSS-induced Caco-2 cells, TEER was elevated while FITC-dextran was diminished in DSS-treated NLRP3^−/−^ Caco-2 cells, thus indicative of repressed cell permeability. However, no significant differences were determined in NLRP3^−/−^ cells, QRXY + NLRP3^−/−^ cells, or TNFα + QRXY + NLRP3^−/−^ cells after DSS treatment (Fig. 5F, G). As indicated by RT-qPCR results, ZO-1, Claudin-1, and Occludin expression were enhanced in DSS-treated NLRP3^−/−^ Caco-2 cells in contrast to DSS-induced Caco-2 cells, while no significant difference was identified among the NLRP3^−/−^ cells, QRXY + NLRP3^−/−^ cells, or TNFα + QRXY + NLRP3^−/−^ cells after DSS treatment (Fig. 5H).

The aforementioned findings demonstrated that NLRP3 knockdown could promote the protective effect of QRXY recipe treatment on DSS-induced Caco-2 cell damage.

### QRXY recipe alleviates UC in mice by inhibiting M1 polarization of macrophages

**Fig. 6:**
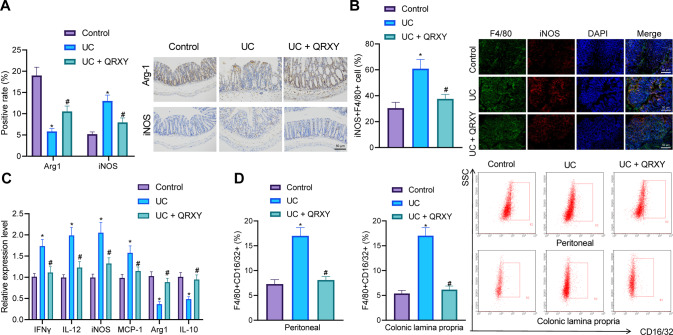
The effect of the QRXY recipe on M1 polarization of macrophages in UC mice. **A** The expression of Arg1 and iNOS protein in the colon tissues of mice after different treatment protocols determined by immunohistochemistry staining. **B** The colon tissues of mice after different treatment protocols were detected by immunofluorescence assay. **C** The mRNA expression of IFNγ, IL-12, iNOS, MCP-1, Arg1, and IL-10 in the colon tissues of mice after different treatment protocols determined by RT-qPCR. **D** F4/80 and CD16/32 positive cells were detected by flow cytometry. *n* = 6 in the control group, and *n* = 9 in other groups. *p < 0.05 compared with that of control mice, ^#^*p* < 0.05 compared with that of UC mice. The cell experiments were conducted three times independently.

We further sought to verify whether macrophage polarization was involved in the anti-inflammatory effect of the QRXY recipe on UC. The expression of macrophage M1 polarization maker inducible nitric oxide synthase (iNOS) in the colon tissues of UC mice was determined using immunohistochemistry, which demonstrated that the iNOS expression was increased, and the expression of macrophage M2 polarization maker Arg1 was reduced, while QRXY recipe treatment could inhibit iNOS expression and elevate the Arg1 expression (Fig. 6A). As reflected by immunofluorescence, the concentration of iNOS^+^ F4/80^+^cells in UC mice was elevated, while QRXY recipe treatment could reduce the concentration of iNOS^+^F4/80^+^ cells, thus indicative of the ability of QRXY recipe treatment to inhibit the polarization of macrophages M1 (Fig. 6B).

Additionally, the RT-qPCR results demonstrated that the expression of M1 macrophage markers IFNγ, IL-12, iNOS, and MCP-1 was elevated in UC mice, while the expression of M2 macrophage markers Arg1 and IL-10 was decreased significantly, and QRXY recipe treatment decreased the expression of IFNγ, IL-12, iNOS, and MCP-1, and increased the expression of Arg1 and IL-10 (Fig. 6C). Peritoneal cells and colonic lamina propria cells were further separated for flow cytometry and our results identified a notably high concentration of F4/80^+^ CD16/32^+^ cells in the peritoneal cells and colonic lamina propria cells of UC mice, while QRXY recipe treatment reduced the concentration of F4/80^+^ CD16/32^+^ cells (Fig. 6D).

Thus, the QRXY recipe relieved UC in mice by inhibiting the M1 polarization of macrophages.

## Discussion

The need of effective outcomes in UC patients remains unmet despite the extensive application of novel therapies and treatment options [16]. Accumulating evidence has suggested the efficiency and safety of traditional Chinese medicine for attenuating UC symptoms [17, 18]. Hence, the current study aimed at investigating the role of QRXY recipes in UC and the involvement of several downstream mechanisms. The experimental data validated the suppressing effects of the QRXY recipe on UC progression through the inactivation of the TNFα/NLRP3/Caspase-1/IL-1β pathway.

Primarily, our findings denoted that the QRXY recipe had the capacity to alleviate UC by repressing the expression of TNFα and M1 polarization of macrophages. Existing literature identified and classified IFNγ, IL-12, iNOS, and MCP-1 as M1 macrophage markers while Arg1 and IL-10 were classified as M2 macrophage markers [19, 20]. Essentially, a positive correlation has been determined between the macrophage’s M1 polarization and the progression of UC [21]. Furthermore, existing research observed decreased levels of M1 macrophage markers with an increase in the levels of M2 macrophage markers, thus eliciting the therapeutic advantages of the Xian-He-Cao-Chang-Yan formula on UC by regulating macrophage polarization [22]. Moreover, to explore the effect of TNFα on UC, TNFα was overexpressed in mice after UC modeling and results elicited that TNFα upregulation could nullify the inhibitory effects of QRXY recipe on ZO-1, Claudin-1, and Occludin contents, thus indicative of exacerbated intestinal mucosal injury, which further exasperated UC in vitro. In consistency with our finding, previous research determined an aberrantly high expression of TNFα in the serum and colonic tissues of patients with active UC [23]. The inhibition of TNFα, which mediates intestinal tract inflammation, has been regarded as a certified therapeutic protocol for UC management [24]. For instance, the hydroalcoholic extract could be adopted in the alleviation of UC by downregulating TNFα [25]. Moreover, the Lizhong Decoction treatment could partly alleviate UC in mice by suppressing TNFα [26]. Besides, ZO-1, Claudin-1, and Occludin have been classified as fundamental tight junction proteins and their repression elicited potential in the alleviation of colitis [27]. Moreover, several tight junction proteins have been identified as vital components in mucosal healing, and thus are implicated as a potential marker of response in the management of UC [28]. Altogether, the preceding findings elicited the protective effect of QRXY on intestinal mucosal injury in UC mice by decreasing TNFα and M1-type polarization of macrophages.

The results from the current study further highlighted that the QRXY recipe attenuated intestinal mucosal injury in UC mice and DSS-induced functional impairment of Caco-2 cells by decreasing the expression of IL-1β through Caspase-1 downregulation *via* reducing TNFα-regulated NLRP3. NLRP3, as a protein complex essential for caspase-1-dependent maturation of the proinflammatory cytokines IL-1β and IL-18, has demonstrated regulation by TNF in murine inflammation-related diseases [29]. A recent study identified as a therapeutic target for DSS-induced UC, 1,25-dihydroxyvitamin D3 by amelioration of NLRP3 could initiate Caspase-1 inactivation [30]. Meantime, an existing study determined the involvement of Caspase-1 downregulation in colon protection initiated by diosgenin in rats with UC [31]. Meanwhile, another study validated that the activation of Caspase-1 increased the production of IL-1β [32]. IL-1β, as a product of the NLRP3 inflammasome, was highly expressed in DSS model mice and Caco-2 cells, and its inhibition could serve as an accomplice in the management of UC [33]. Moreover, TNFα can induce Caspase-1 activation in an NLRP3 inflammasome-independent manner [34]. Additionally, weakened expression of TNFα, IL-1β, NLRP3, and Caspase-1 was identified due to ginsenoside Rk3, which extended a protective effect on intestinal barrier function and ultimately alleviated DSS-induced UC [35]. Intriguingly, anti-TNFα has been indicated as a developmental advance for UC treatment [36]. An existing study highlighted the ability of a reduced TNFα expression to alleviate UC by optimizing intestinal microbiota [37]. Conclusively, our results elicited that the inactivation of the TNFα/NLRP3/Caspase-1/IL-1β pathway by QRXY recipe could serve as a therapeutic modality for intestinal mucosal injury in UC mice and DSS-induced functional impairment of Caco-2 cells.

**Fig. 7:**
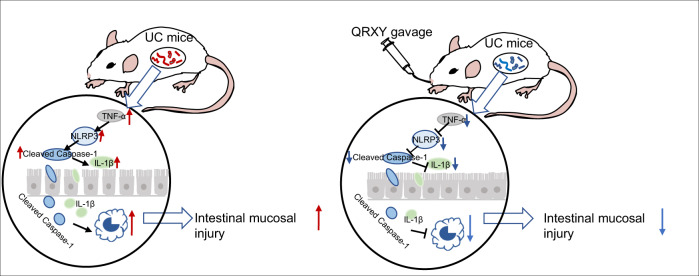
The molecular mechanism of QRXY recipe inhibiting TNFα/NLRP3/Caspase-1/IL-1β axis and macrophage M1 polarization to reduce intestinal mucosal damage in UC mice.

To conclude, the aforementioned findings implicated that the QRXY recipe reduced intestinal mucosal damage by inhibiting the TNFα/NLRP3/Caspase-1/IL-1β signal pathway and macrophage M1 polarization, protected intestinal epithelial cells, and fundamentally alleviated UC (Fig. 7). The current study revealed the potential molecular mechanism of QRXY recipe in the treatment of UC as a novel insight for the development of new drugs and clinical application. However, further investigation is warranted for the identification of alternative molecular mechanisms for superior clinical outcomes in UC management.

## Materials and methods

### Ethics statement

The current study was performed with the approval of the Ethics Committee of The Affiliated Hospital of the Nanjing University of Chinese Medicine. All animal experiments were strictly designed and performed in accordance with the recommendations of the Guide for the Care and Use of Laboratory Animals published by the *National Institutes of Health*. Extensive measures were taken to ensure minimal suffering for the included animals.

### Network pharmacology and in silico analysis

The potential targets from ten types of herbs (Coptis 3 g, parched white peony root 10 g, parched angelica 10 g, simmered radix aucklandiae 10 g, sanguisorba officinalis 15 g, lithospermum15 g, agrimonia pilosa ledeb 15 g, parched atractylodes macrocephala koidz 10 g, poria cocos 15 g, radix glycyrrhizae preparata 5 g) of the QRXY recipe were identified using the SymMap database. The UC-related microarray dataset GSE53835 was obtained through the gene expression omnibus (GEO) database, the microarray contained platform annotation file GPL1261, and the sample grouping information is presented in Supplementary Table 2. The DEGs were analyzed using the “limma” package of R language with the threshold set as |log2 fold change (FC) | > 1, *p* < 0.05. An intersection between the potential targets and the DEGs in the UC-related microarray dataset GSE53835 was identified using the jvenn online tool to predict the potential regulators of the QRXY recipe to subsequently attenuate colitis. The expression heat map of candidate genes in the UC-related microarray dataset GSE53835 was plotted using the “pheatmap” package of R language. KEGG pathway enrichment analysis of upregulated genes and downregulated genes was performed by the means of NetworkAnalyst tool. A combination of the coexpedia database and BioGRID database were used to predict the interaction genes among the factors for subsequent prediction of the downstream regulatory factors of candidate genes. The GeneMANIA database was used to analyze the function and the coexpression relationship of the candidate genes.

### Mice model with UC induced by DSS

A total of 60 C57BL/6 mice aged 7–8 weeks were housed in strict accordance with the specific pathogen-free (SPF) feeding protocols. The mice were housed in a relatively noise-free isolation room at 22–24 °C with a humidity of 50–60% and alternating dark–light circles for 12 h. The mouse food and padding were all disinfected by high-temperature treatment, while the drinking water was disinfected by high-temperature and high-pressure treatment. The padding was changed once weekly, while the food and drinking water were replenished daily. The full-length cDNA sequence in the TNFα coding region was inserted into the psc adeno-associated viruses (AAV)-MCS to construct the pscAAV-TNFα plasmid. The AAVs were prepared by the AAV-Helper-Free system Cell Biolab (Cell Biolabs, San Diego, CA, USA). After fasting for 12 h, the mice were anesthetized using an isoflurane vaporizer, and a 4 cm soft catheter was inserted into the anus of mice. The constructed AAV2-control (AAV-control) or AAV2-TNFα (AAV-TNFα) [5 × 10^10^ μg dissolved in 100 μL phosphate-buffered saline (PBS)] were clustered into mice through a tube, after which the mice were kept in an upside-down position for 1 min. After recovering from anesthesia, the mice were fed for another two weeks prior to subsequent experiments. The mice were fed with 3% (w/v) DSS (60316ES60, Yeasen, Shanghai, China) dissolved in drinking water for 7 consecutive days for a total of 5 weeks to induce acute UC.

The successfully established UC mice were treated by QRXY (*n* = 9), AVV-Control (*n* = 9), AVV-Control + QRXY (*n* = 9), AVV-TNFα + QRXY (*n* = 9), while the untreated UC mice were regarded as control (*n* = 6). QRXY was administrated via gavage 30 min before modeling (daily dose of 1.62 g crude drug/kg, dissolved in drinking water). The body weight of mice after different treatment protocols was documented every day, and the mice were euthanized on the seventh day. The peripheral blood was isolated from the eyes of the mice after anesthesia, while the plasma was separated by centrifugation at 4000 rpm. After dissection, the jejunum and colon were taken for subsequent analysis.

### DAI score

During administration and modeling, the alterations of mice weight, fecal character, and hemafecia were observed and documented daily and calculated according to the DAI scoring standard, DAI = (weight loss score + fecal character score + hemafecia score)/3. DAI scoring standards were as follows: 0 points: no weight loss, normal fecal character, and no hemafecia; 1 point: 1–5% weight loss, loose stools, and stool occult blood; 2 points: 5–10% weight loss, loose stools, and stool occult blood; 3 points: 10–15% weight loss, loose stools, and bloody stools. 4 points: over 15% weight loss, loose stools, and bloody stools.

### H&E staining

Colon tissues were fixed in 10% neutral buffered formalin (G2161, Solarbio, Beijing, China) at 4 °C for 24 h, dehydrated, immersed in wax, embedded, and then sliced. The slices were deparaffinized using xylene for 5–10 min, and subsequently deparaffinized for another 5–10 min with fresh xylene. Next, the slices were subjected to treatment with absolute ethanol for 5 min, 90% ethanol for 2 min, 80% ethanol for 2 min, 70% ethanol for 2 min, distilled water for 2 min, and stained with the hematoxylin staining solution for 5–10 min. The excess staining solution was rinsed with deionized water for 10 min. Next, slices were stained with the eosin staining solution for 30 s–2 min. The slices were then dehydrated with 70% ethanol for 10 s, 80% ethanol for 10 s, 90% ethanol for 10 s, and absolute ethanol for 10 s, cleared using xylene for 5 min, and again for another 5 min with fresh xylene. Subsequently, slices were mounted with neutral gum, while the observations were documented under an inverted microscope (IX73, Olympus, Tokyo, Japan).

According to the criteria of Dieleman, the severity of the lesion was scored based on the depth and extent of the ulcer as well as the degree of inflammation, and the depth of the lesion, the scoring was as follows: 0 points, no disease; 1 point, the invasion of salina layer by the lesion; 2 points, invasion of the sub-anvil membrane by the lesion; 3 points, invasion of the muscle layer by the lesion; 4 points, invasion of the serosal layer by the lesion. The extent of lesions was scored as follows: 0 points, no lesions. 1 point, 0–25%; 2 points, 26–50%; 3 points: 51–75%; 4 points, >75%. The degree of inflammation was scored as follows: 0 points, no inflammation; 1 point, mild inflammation; 2 points, moderate inflammation; 3 points, severe inflammation.

### Immunohistochemistry

Mouse colon tissues were embedded in paraffin and sliced with an ultra-thin microtome. Slices were then deparaffinized with xylene, rehydrated with gradient alcohol, and incubated with 3% H_2_O_2_ to block activity of endogenous peroxidase. Subsequently, the slices were boiled in 10 mM sodium citrate (pH 6.0) for 30 min, then blocked with 10% normal goat serum for 15 min, and incubated with the corresponding Abcam (Cambridge, UK)-purchased primary rabbit antibodies to NLRP3 (ab214185, 1: 100), arginase 1 (Arg1, ab233548, 1: 2000), iNOS (ab115819, 1: 100) overnight in a damp room at 4 °C. The following day, slices were incubated with the corresponding secondary antibodies goat anti-rabbit horseradish peroxidase (HRP)-labeled immunoglobulin G (IgG) (ab205718, 1: 2000) for 1 h at room temperature, and a 2,4-diaminobutyric acid (DAB) kit (Invitrogen, Carlsbad, CA, USA) was used for immunoreactivity detection.

### Immunofluorescence

Mouse colon tissues were fixed using 4% paraformaldehyde and then divided into slices with a thickness of 40 μm using a vibrating knife (VT1200s, Leica, Wetzlar, Germany). After antigen retrieval with sodium citrate buffer, the slices were permeabilized with 0.1% Triton X-100 and blocked with the addition of 10% normal donkey serum for 1 h. Then slices were incubated overnight at 4 °C with primary antibodies rabbit anti-F4/F80 (ab6640, 1: 200), rabbit anti-iNOS (ab178945, 1: 500), mouse anti-Claudin (ab242370, 1: 500), rabbit anti-Occludin (ab216327, 1: 100), and rabbit anti-ZO-1 (ab221547, 1: 100). The slices were incubated with the secondary antibody (A32766/A-21203/A-21206/A32754, 1: 500, Thermo Fisher) coupled with Alexa Fluor 488/594 for 1 h. Subsequently, the slices were stained with 4’,6-Diamidino-2-Phenylindole (DAPI) for 10 min, and documented under a fluorescence microscope (Olympus).

### Collection of peritoneal macrophages

Peritoneal macrophages were isolated from mice after an intraperitoneal injection with 4 mL Roswell park memorial institute (RPMI)-1640 containing 5% fetal bovine serum (FBS) and 0.5 mM ethylene diamine tetraacetic acid (EDTA). Some cells were reserved for immediate analysis of the frequency of M1 macrophages, while the remaining cells were centrifuged at 300 g for 10 min and cultured in RPMI-1640 medium at 37 °C.

### Isolation of lamina propria cells

The dissected colon tissues were rinsed with penicillin/streptomycin-supplemented PBS and minced, incubated in RPMI-1640 medium (Gibco, Grand Island, NY, USA) containing a combination of 3% FBS (Gibco), 0.5 mM dithiothreitol (Sigma-Aldrich, St. Louis, MO, USA) and 5 mM EDTA (Sigma-Aldrich), then treated with antibiotics at 37 °C for 30 min to eliminate the epithelial cell layer. Next, the tissues were incubated with RPMI-1640 medium containing equivalent amounts of 0.5% Collagenase D (Roche, Basel, Switzerland) and 0.05% DNase (Roche) at 37 °C for 30 min. The single-cell suspension was prepared from the derived cells, which were filtered through a 70 µm filter and separated for flow cytometry.

### TUNEL staining

Cell apoptosis was detected using a TUNEL kit in strict accordance with the provided instructions (Roche). The colon tissues of mice in each group were embedded in conventional paraffin and sliced. Slices were baked in a 65 °C oven for 1–2 h, dewaxed, hydrated, supplemented with Proteinase K working solution, and digested in a dark humidified chamber at 37 °C for 30 min. Next, the slices were incubated with 50 μL of the TUNEL reaction mixture for 60 min in a dark humidified box at 37 °C. Slices were mounted with an anti-fluorescence quencher and observed under a fluorescence microscope (Olympus) at the excitation wavelength of 488 nm.

### Construction of NLRP3 knockout cell model

Caco-2 cell line was purchased from Procell (CL-0050, Wuhan, Hubei, China) and cultured in DMEM containing a combination of 10% FBS (Gibco), 1% non-essential amino acids (Gibco), 100 U/mL penicillin (Gibco) and 100 μg/mL streptomycin at 37 °C with 5% CO_2_. The NLRP3 knockout Caco-2 cell line was constructed based on the CRISPR/Cas9 technology, which was completed by the Technology Center of Chongqing Weston Biomedical Technology Co., Ltd. (Chongqing, China). The sgRNA sequence was derived from the Genscript website (https://www.genscript.com/), and the NLRP3 sgRNA sequence was as follows: 5’-CGAAGCAGCACTCATGCGAG-3’.

### Cell treatment

Upon attaining 80–90% cell confluence, the Caco-2 cells (RRID: CVCL_0025) were digested with 0.25% trypsin containing EDTA, passaged, and seeded on Transwell culture plates (3413, Corning Incorporated, NY, USA, pore size 0.4 μm, effective area of 0.33 cm^2^) at a density of 5 × 10^4^ cells/chamber. The upper layer was supplemented with 0.5 mL of cell suspension, while the lower layer was supplemented with 1.5 mL of culture medium. The medium was replaced on alternate days. After a period of 7 days, the medium was replaced every day. The cells were cultured for a total of 21 days after which the formation of a dense monolayer was evident. The cell layer was arranged in a monolayer, merged with each other, with clear boundaries and clear tight connections between the cells. The Caco-2 cells after UC modeling were treated by QRXY, TNFα + QRXY, or without any treatment, and the Caco-2 cells without any treatments were set as control. NLRP3^−/−^ Caco-2 cells: NLRP3 was knocked out in the Caco-2 cells after UC modeling, QRXY + NLRP3^−/−^, TNFα + QRXY + NLRP3^−/−^, or without any treatment, and NLRP3^−/−^ Caco-2 cells were set as blank control. DSS treatment: Caco-2 cell monolayers were cultured without serum for 24 h, and then treated with 2% DSS (w/v) in the basolateral chamber of Transwell for 24 h. For TNFα treatment, Caco-2 cells were rinsed three times with sterile PBS (pH 6.7), after which the monolayer was treated with 10 ng/mL TNFα (Sigma-Aldrich) for 24 h.

### Preparation of QRXY recipe

The aforementioned QRXY recipe was weighed according to the adult dosage (108 g), soaked in the extraction tank for 60 min, decocted 2 times with the preparation of a solution by the combination of liquid medicines, evaporated in the rotary evaporator, concentrated under low pressure, and homogenized into powder form in a vacuum drying box. Next, 41.56 g of QRXY ointment was taken, which was 38.5% of the extract, and 0.15 g of fine drug powder was dissolved in 15 mL of sterilized PBS, at a concentration of 15 g/L, filtered, sterilized, aliquoted, and stored at −20 °C. An appropriate amount was taken for dosing and diluted to a final concentration of 10 μg/mL.

### CCK-8 assay

After different treatment protocols, cell viability was measured using a CCK-8 kit (GK10001, GLPBIO, Montclair, CA, USA) as per the provided instructions. CCK-8 reagent (10 μL) was supplemented to each well and mixed before 1-h incubation, after which the optical density (OD) value was measured at 450 nm. The experiments were conducted three times independently.

### Determination of LDH

The LDH release levels in the supernatant of cultured cells were measured in the light of the protocols of an LDH assay kit (C0016, Beyotime, Shanghai, China).

### Detection of cell monolayer integrity and permeability

The integrity and permeability of the cell monolayer were determined by estimation of the TEER and FITC-dextran (Sigma-Aldrich) permeability. After the establishment of tight junctions of intestinal epithelial cells, the transmembrane resistance was measured using a cell resistance meter (MERS00002, Merck Millipore, Billerica, MA, USA), with repeated measurements on alternate days for a comprehensive understanding of the dynamic formation process of monolayer integrity. To guarantee the stability and accuracy of the value, the vigorous measurement process was conducted at 37 °C. After the removal of the culture plate, it was equilibrated on the ultra-clean table for 0.5 h. Three different directions were chosen in each Transwell culture plate for repetitive measurement during the assessment. The TEER was calculated as the average value × the membrane area of the Transwell culture plate. As the Transwell membrane itself has a certain TEER value, the actual TEER value should be determined by subtraction of the TEER value of the blank control from the measured value.

After the successful establishment of tight junctions of intestinal epithelial cells in the Caco-2 cell line, the Transwell plate was slowly rinsed twice with Hank’s balanced salt solution (HBSS) which had been preheated to 37 °C. Afterward, 0.5 mL of 200 μg/mL of fluorescent yellow solution was supplemented to the apical chamber of the Transwell plate, and 1.5 mL of HBSS buffer was supplemented to the basolateral chamber. After 2-h incubation, 100 μL solution was taken from both the apical and basolateral chambers of the Transwell chambers. A multi-functional microplate reader (Multiskan FC, Thermo Fisher Scientific, Waltham, MA, USA) was applied to evaluate the fluorescence intensity, with the calculation of the fluorescence permeability and the apparent permeability coefficient of transmembrane transport (Papp). The calculation formula was as follows: Papp = Δ*Q*/(Δ*t* *A* Co) (cm s^−1^), where Δ*Q* is the amount of fluorescence transport within Δ*t*, *A* (cm^2^) is the membrane area, and Co is the initial concentration of fluorescence on the top side of Caco-2 cells.

### Flow cytometry

The medium of each group from the cell culture plate was transferred to a 15 mL conical tube and placed on ice. The cells were supplemented with 0.5 mL of 0.25% trypsin and incubated until cell detachment on the culture plate wall was evident under the microscope. The cells were tapped gently and continuously to detach cells from the culture plate wall completely. The cells were resuspended in the previously collected medium to attain a density of approximately 1 × 10^6^ cells/mL. A total of 0.5 mL of cell suspension (5 × 10^5^ cells) was transferred from the cell culture plate to a clean centrifuge tube and added to the staining solution. Next, the cells were resuspended with 0.5 mL of pre-chilled 1× binding buffer, and incubated with a combination of 5 μL Annexin V-FITC and 10 μL propidium iodide (PI) in the dark for 15 min, after which a flow cytometer (FACSVerse/Calibur/AriaIISORP, BD Bioscience) was used for detection and analysis. The reagents were provided by Beyotime.

Peritoneal macrophages and lamina propria cells were stained with FITC rat anti-mouse F4/80 (BD Bioscience) and PE anti-mouse CD16/32 (BD Bioscience) at 4 °C for 30 min. F4/80 was used as a macrophage marker, while the F4/80^+^CD16/32^+^ cells were classified as M1 macrophages. FACS Canto II cytometer (BD Bioscience) was used for data collection, and the FACSDiva (BD) and FlowJo software packages (TreeStar, Ashland, OR, USA) were used for data analysis.

### RT-qPCR

The total RNA content was extracted from the tissues using a Trizol kit (16096020, Invitrogen). For mRNA detection, the reverse transcription kit (RR047A, Takara, Tokyo, Japan) was employed to perform reverse transcription for the preparation of cDNA. The sample was subjected to RT-qPCR in a real-time fluorescent quantitative PCR machine (ABI 7500, ABI, Foster City, CA, USA), followed by a subsequent regimen of RT-qPCR reaction. RT-qPCR was performed as per the provided manuals of the TaqMan Gene Expression Assays protocol (Applied Biosystems, Foster City, CA, USA). The primer design was as presented in Supplementary Table 3. The fold changes were calculated based on the 2^−^^ΔΔCt^ method.

### Western blot analysis

The total protein content was extracted from cells after different treatment protocols using the radioimmunoprecipitation assay (RIPA) lysis buffer (Beyotime) before estimation of the protein concentration using a bicinchoninic acid (BCA) kit (20201ES76, Yeasen). Subsequent to separation by polyacrylamide gel electrophoresis, the protein was electro-blotted to a polyvinylidene fluoride membrane (IPVH85R, Millipore) by the wet transfer method, which subsequently underwent 1-h 5% bovine serum albumin sealing at ambient temperature. The membrane was probed with primary rabbit antibodies against NLRP3 (ab214185, 1: 1000, Abcam), Cleaved Caspase-1 (#4199, 1: 1000, CST, Danvers, MA, USA), mouse IL-1β (#12242, 1: 1000, CST), and β-actin (ab8226, 1: 3000, CST) overnight at 4 °C. The membrane was supplemented with the corresponding HRP-labeled goat anti-rabbit IgG (ab6721, 1: 5000, Abcam) or goat anti-mouse IgG (ab6789, 1: 5000, Abcam) for 1 h at ambient temperature. The membrane was supplemented with the luminescent solution prior to development. Protein quantitative analysis was implemented using the ImageJ software (National Institutes of Health, Bethesda, Maryland, USA), and the protein quantitative analysis was estimated as the ratio between the gray value of each protein and the gray ratio of the internal reference β-actin.

### ELISA

The supernatant from the mouse colon tissues or Caco-2 cells was collected for content detection of relevant inflammatory factors as per the provided protocols of the ELISA kits. The kits used for supernatant isolation of mouse colon explants were as follows: mouse interleukin-6 (IL-6) ELISA kit (ab100713, Abcam), mouse interleukin-8 (IL-8) ELISA kit (ml063162, LiankeBio, Hangzhou, China), mouse interleukin-1β (IL-1β) ELISA kit (ab197742, Abcam) and mouse TNFα ELISA kit (ab208348, Abcam). The kits used for supernatant isolation of Caco-2 cells were as follows: human IL-6 ELISA kit (ab178013, Abcam) and human IL-8 ELISA kit (ab214030, Abcam).

### Statistical analysis

All data were analyzed using the GraphPad Prism statistical software (Version X, La Jolla, CA, USA). The measurement data were described as mean ± standard deviation. Data between the two groups were compared using the unpaired *t*-test. Differences among multiple groups were statistically analyzed employing one-way analysis of variance (ANOVA) and Tukey’s post hoc test. Statistical analysis in relation to time-based measurements within each group was conducted using repeated measures ANOVA, followed by Tukey’s post hoc test. In all statistical references, a value of *p* < 0.05 was statistically significant.

## Supplementary information

Supplementary Figure 1

Supplementary Tables

Original Data File

## Data Availability

All data generated or analyzed during this study are included in this article [and/or] its supplementary material files. Further inquiries can be directed to the corresponding authors.
