# Supplementary figures and images for: Qingre Xingyu recipe exerts inhibiting effects on ulcerative colitis development by inhibiting TNFα/NLRP3/Caspase-1/IL-1β pathway and macrophage M1 polarization
Source: Cell Death Discov. 2023 Mar 8;9:84. doi: 10.1038/s41420-023-01361-w (PMC9995513; doi:10.1038/s41420-023-01361-w)

**Supplementary Fig. 1** The KEGG enrichment network of upregulated genes.


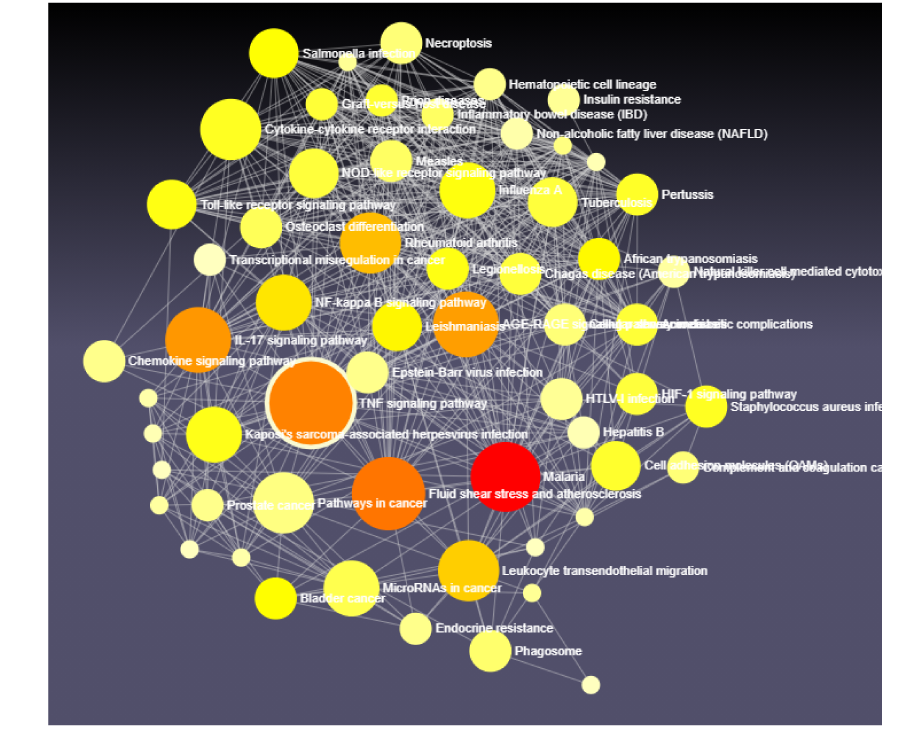

Supplement: Supplementary file 1 — Supplementary Figure 1 [file 41420_2023_1361_MOESM1_ESM.docx]

**DSS**


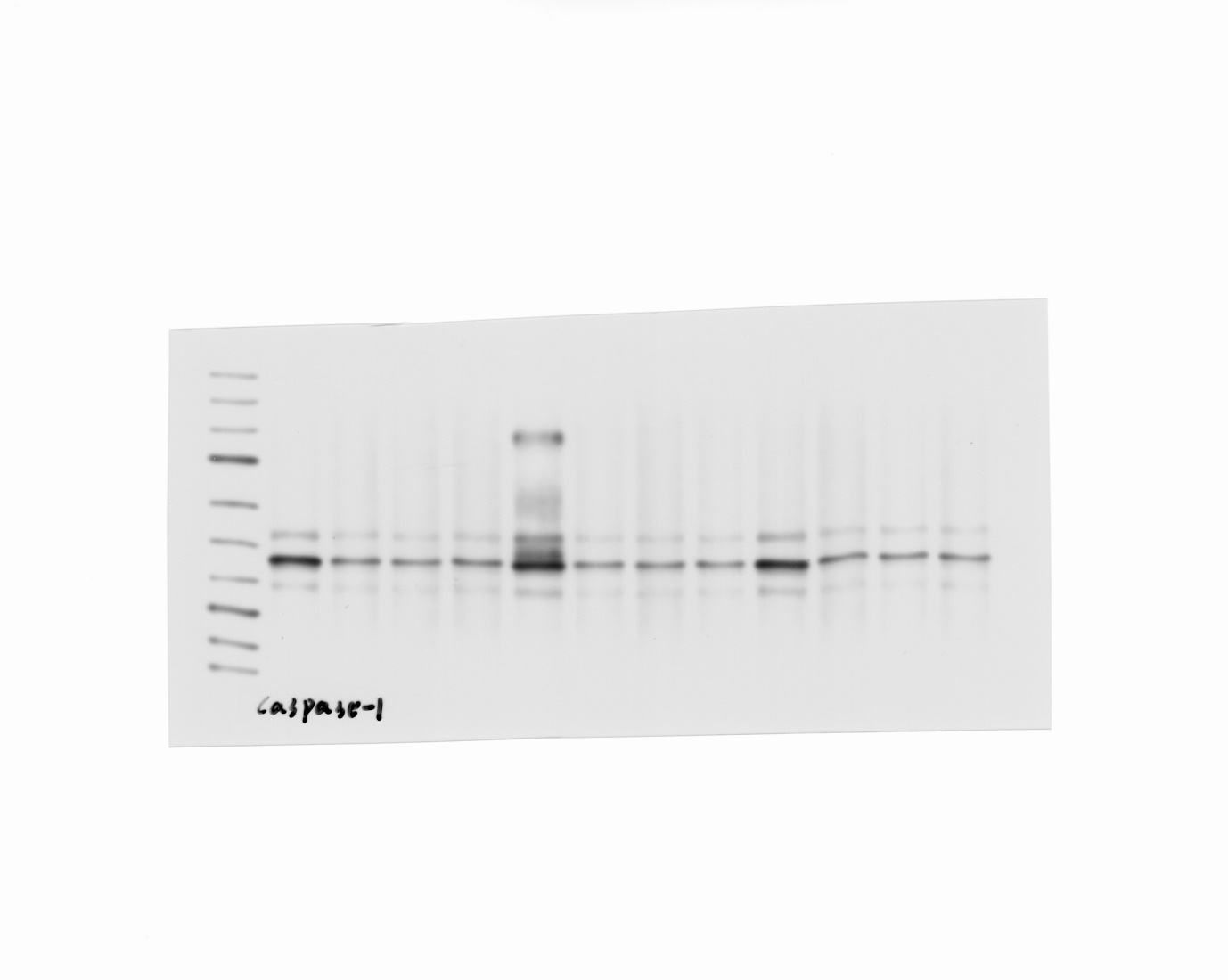

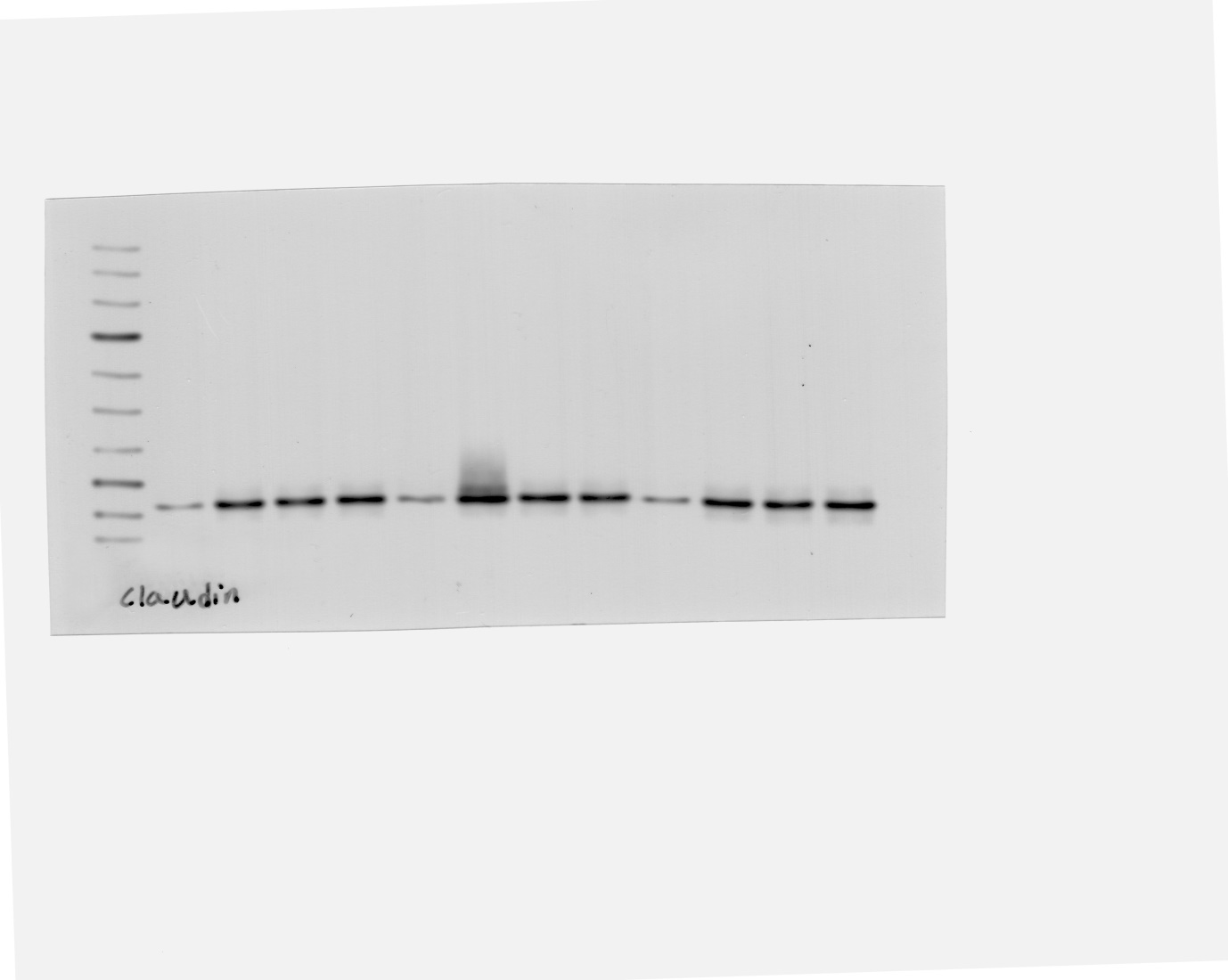

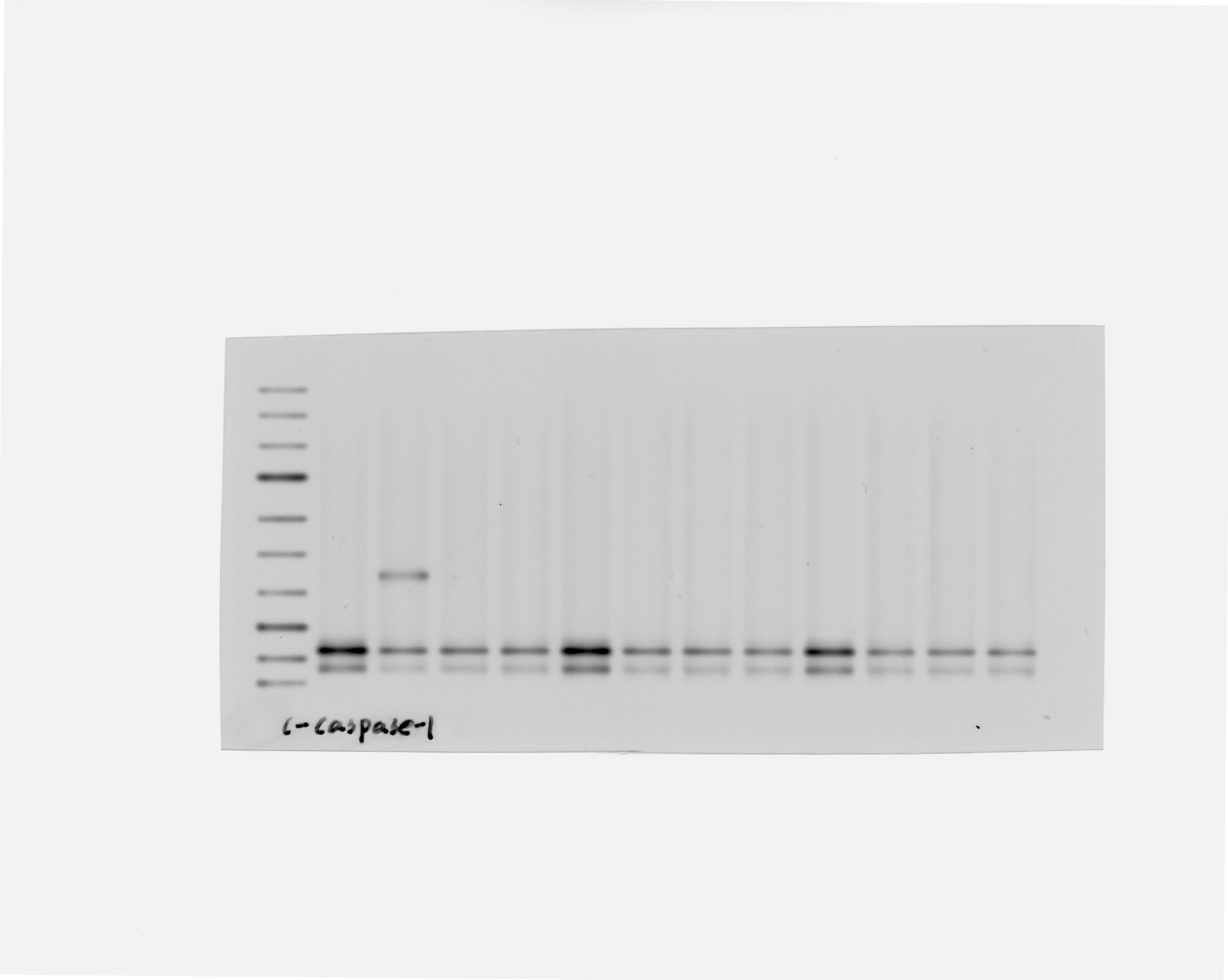

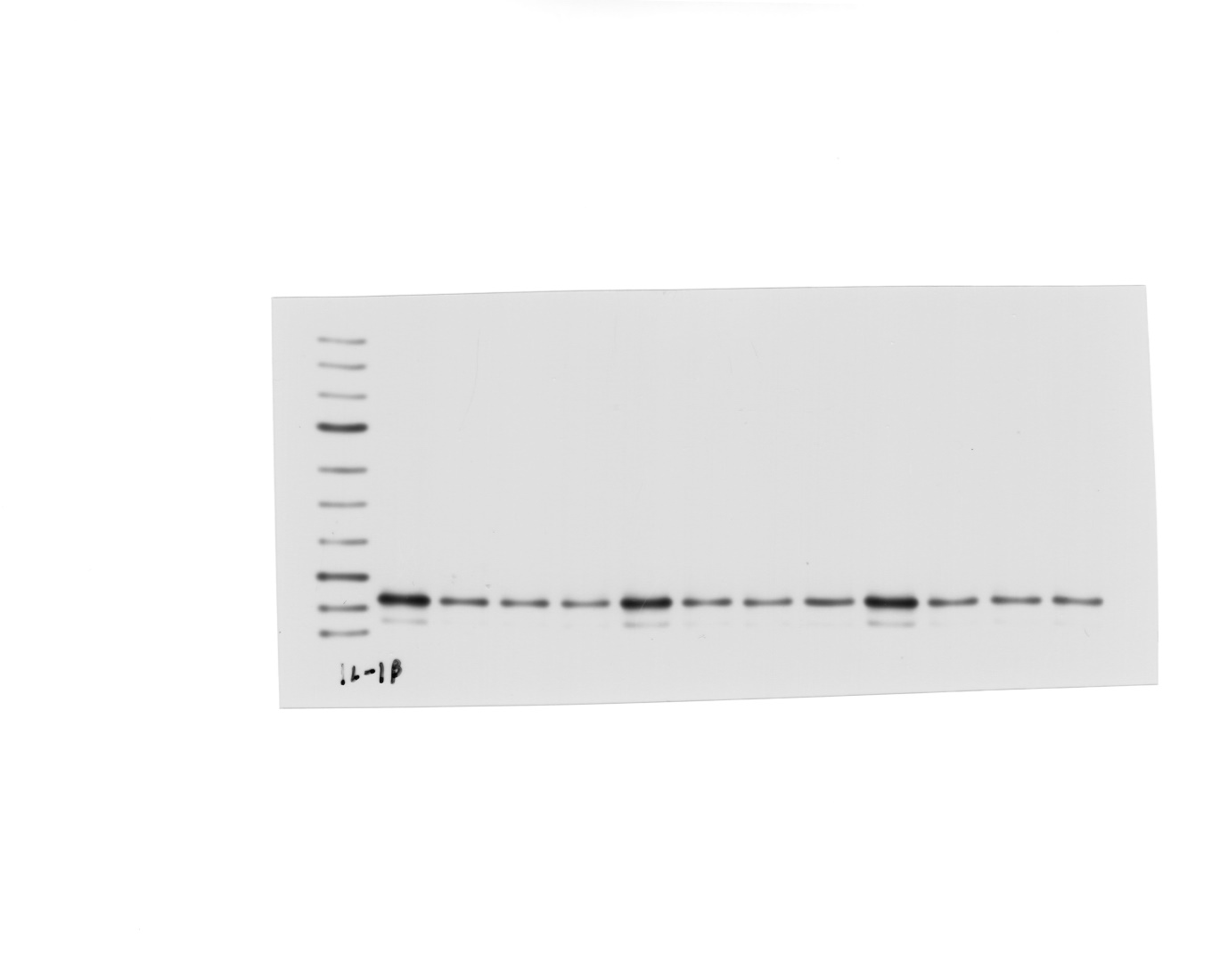

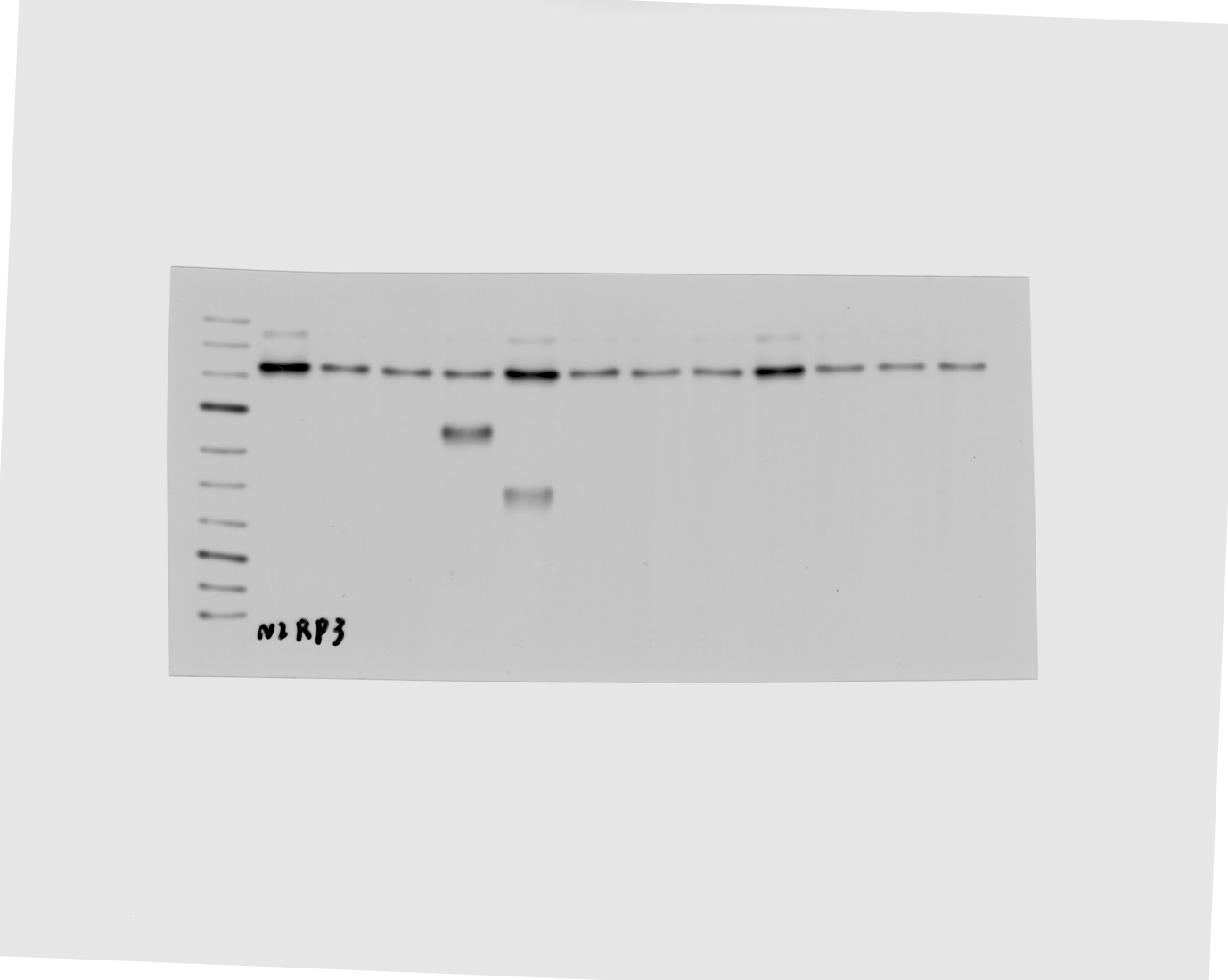

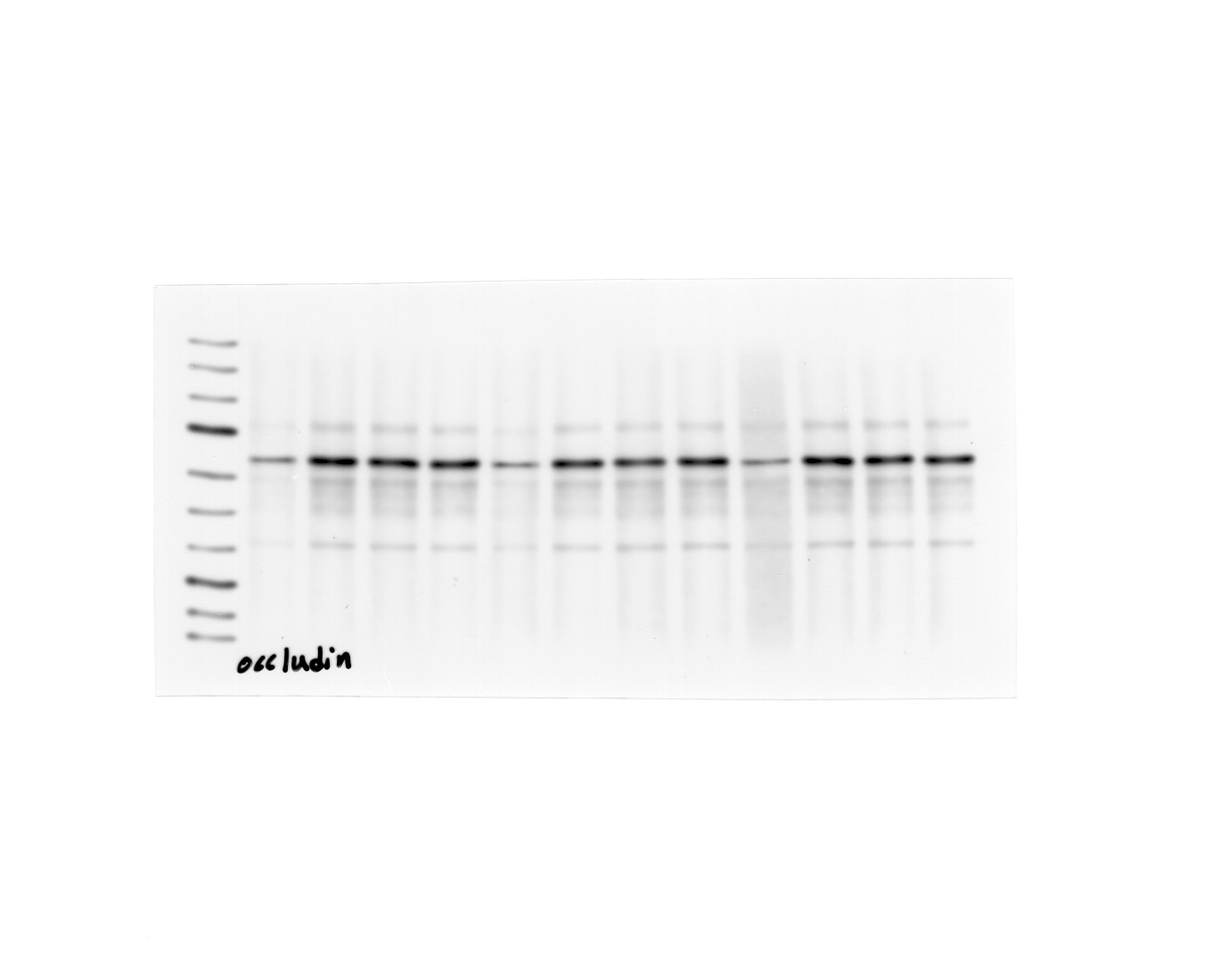

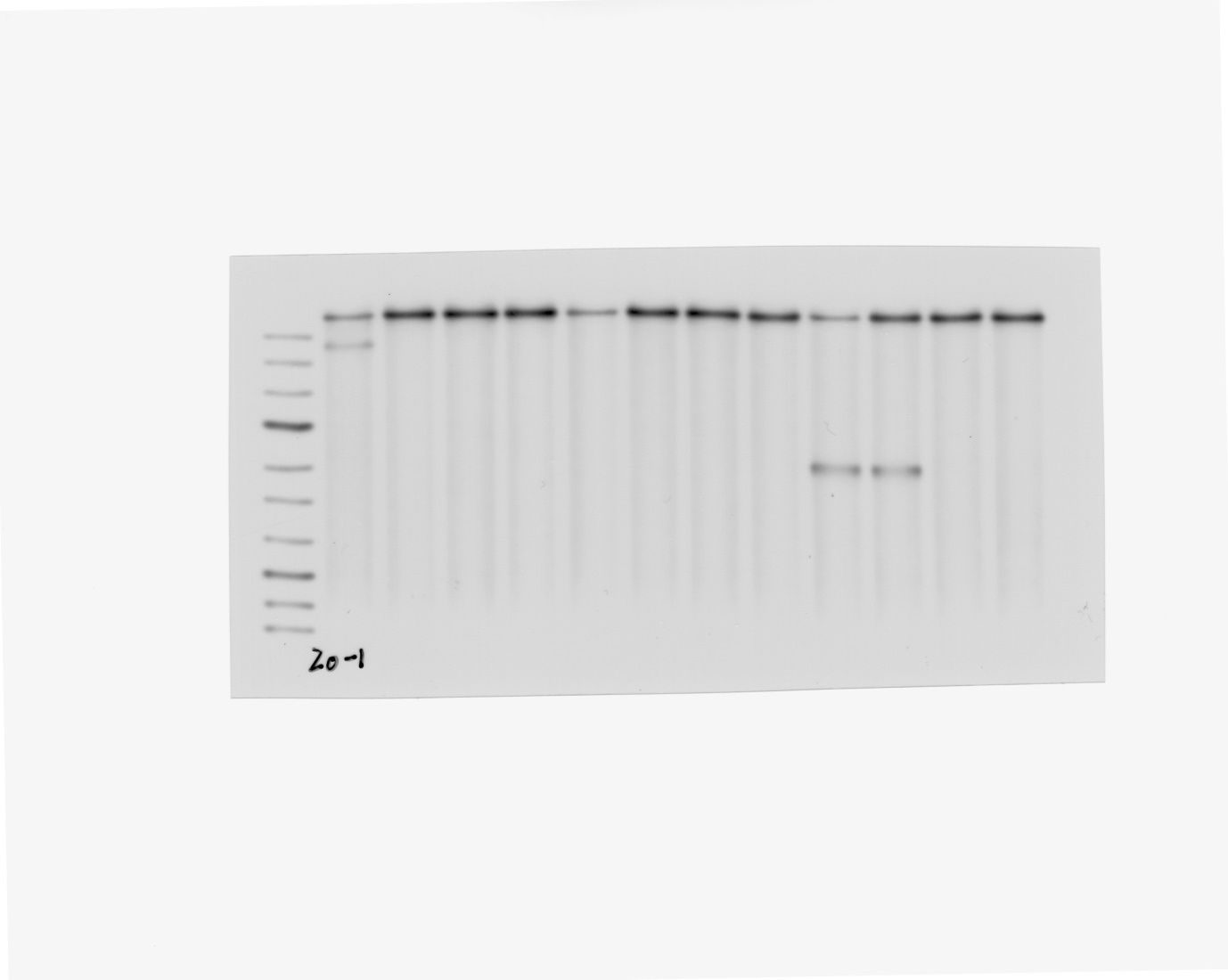

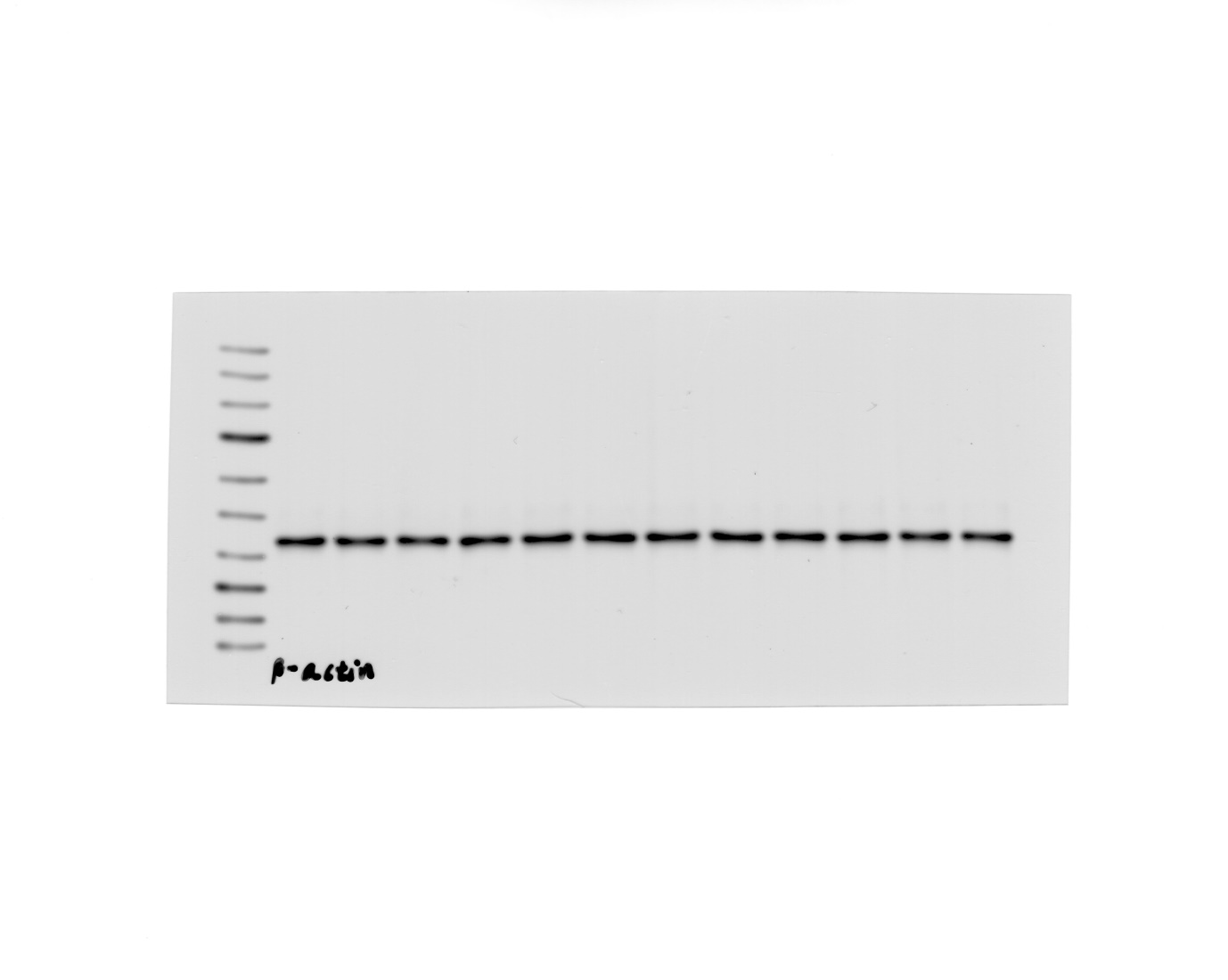


**NLRP3**


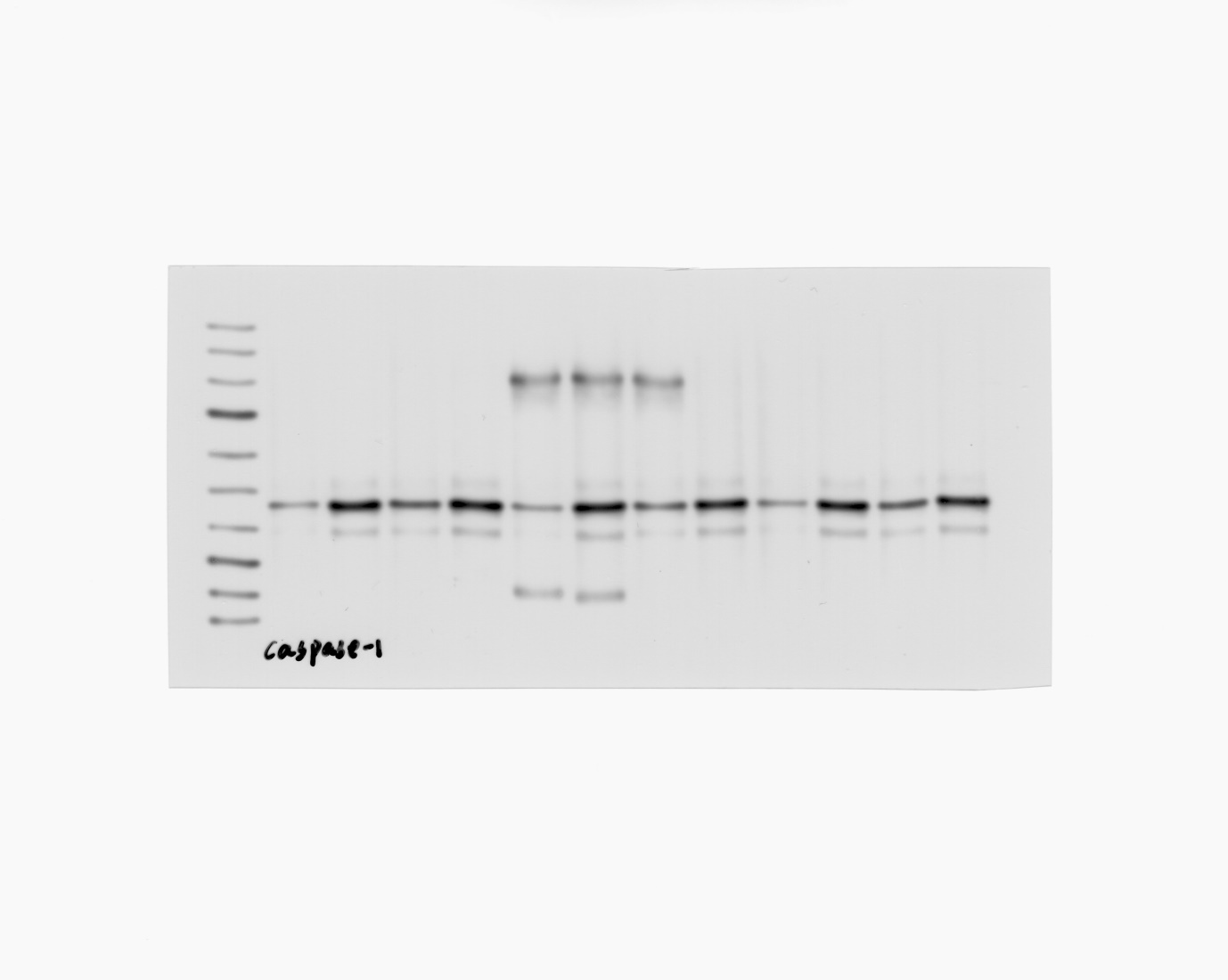

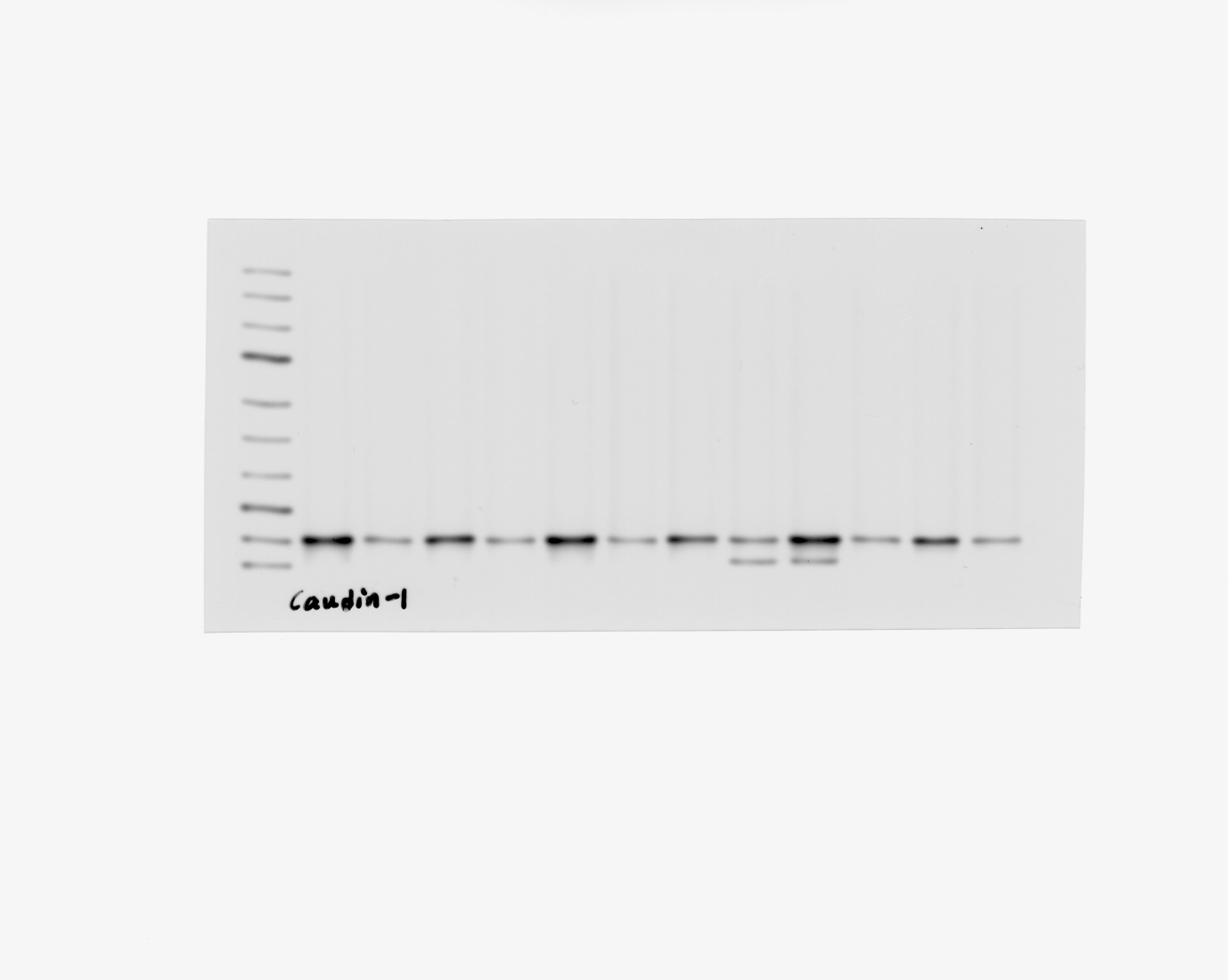

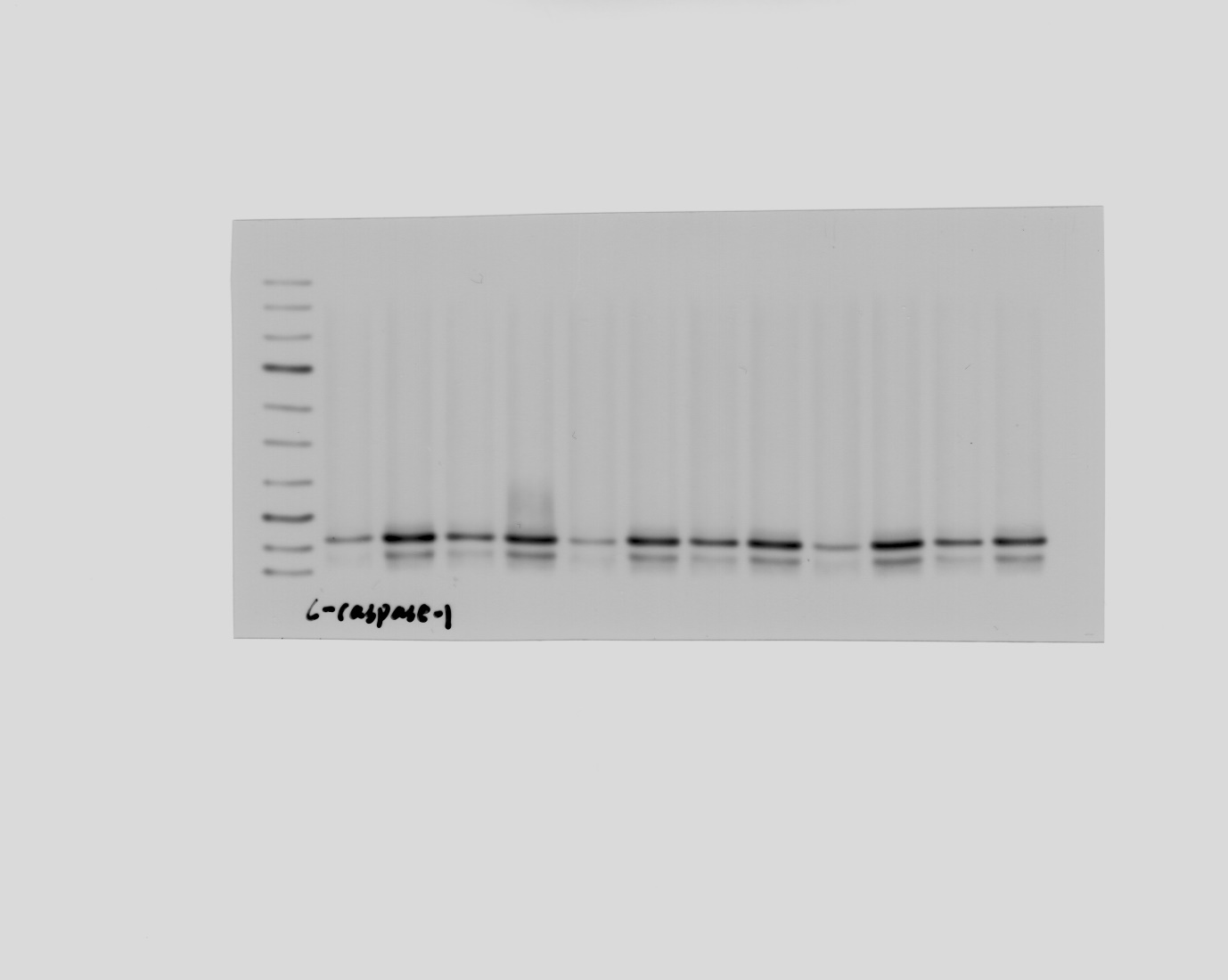

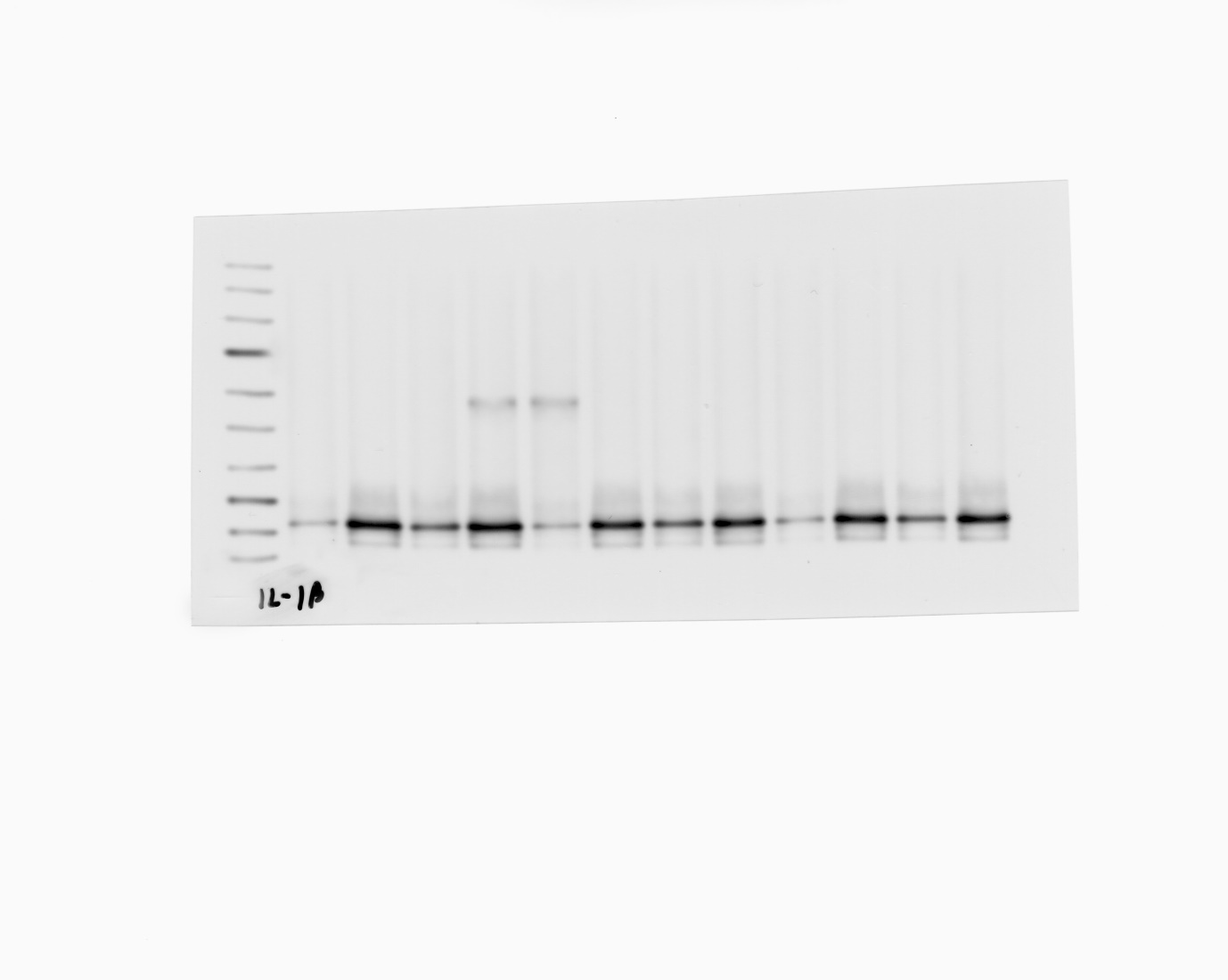

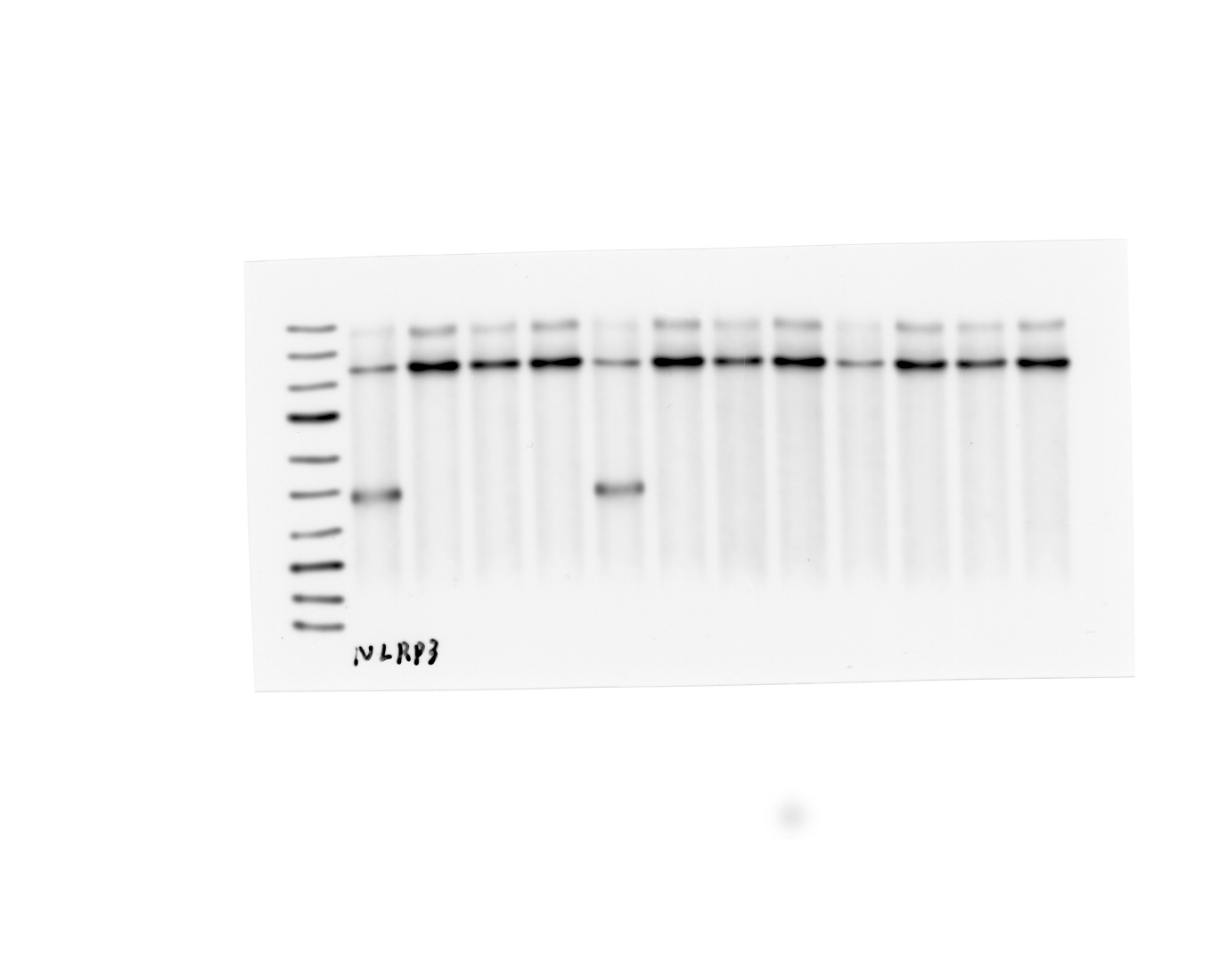

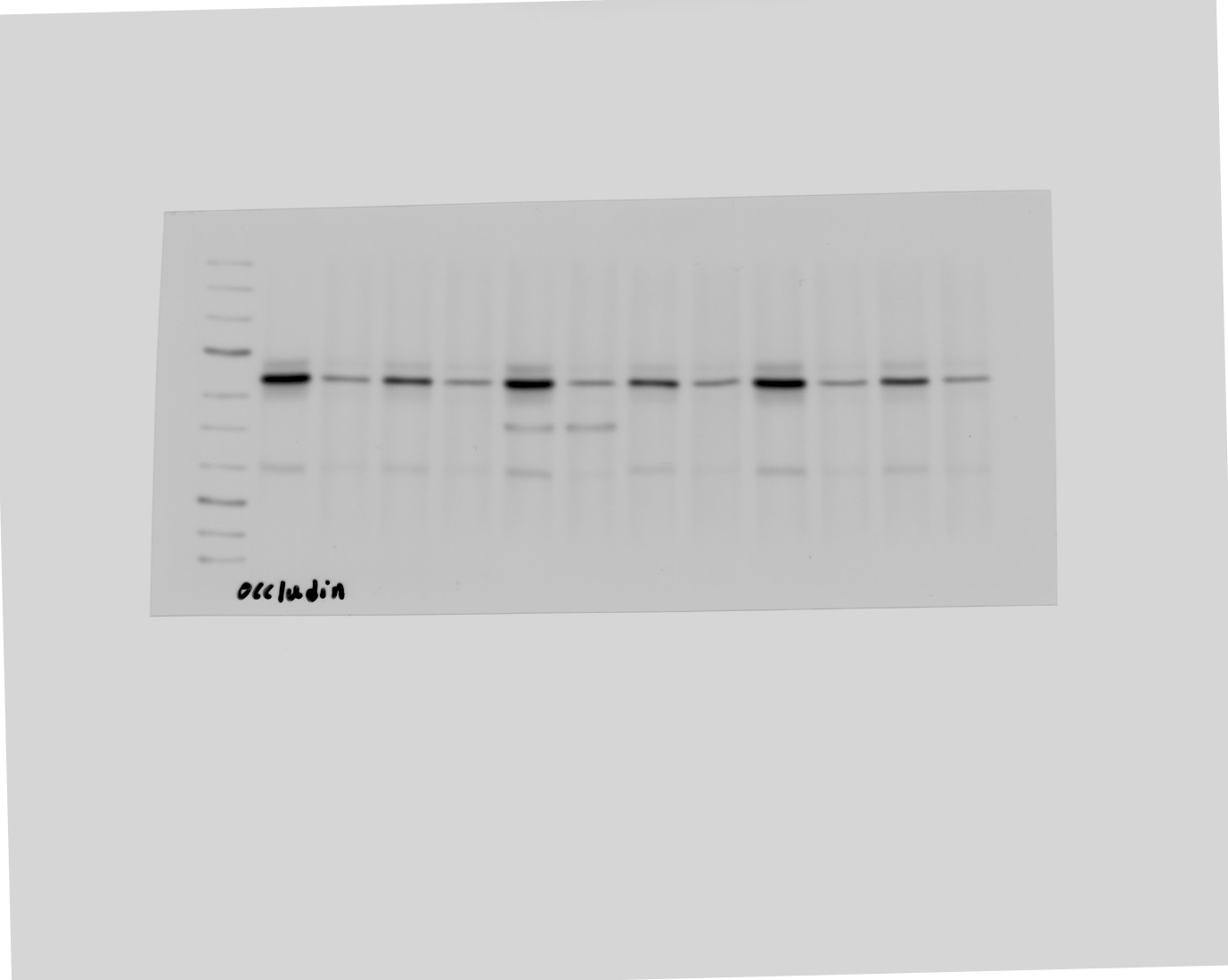

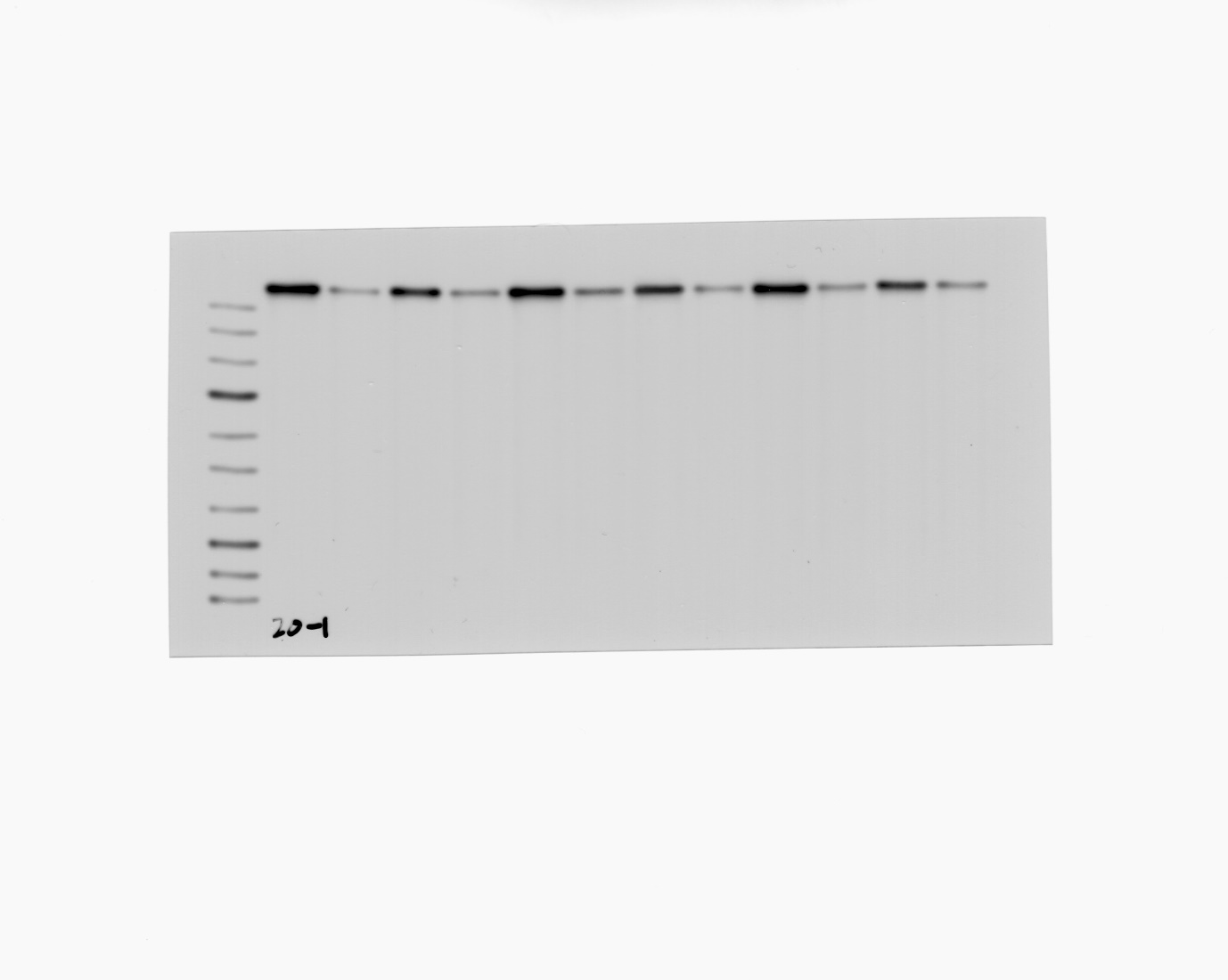

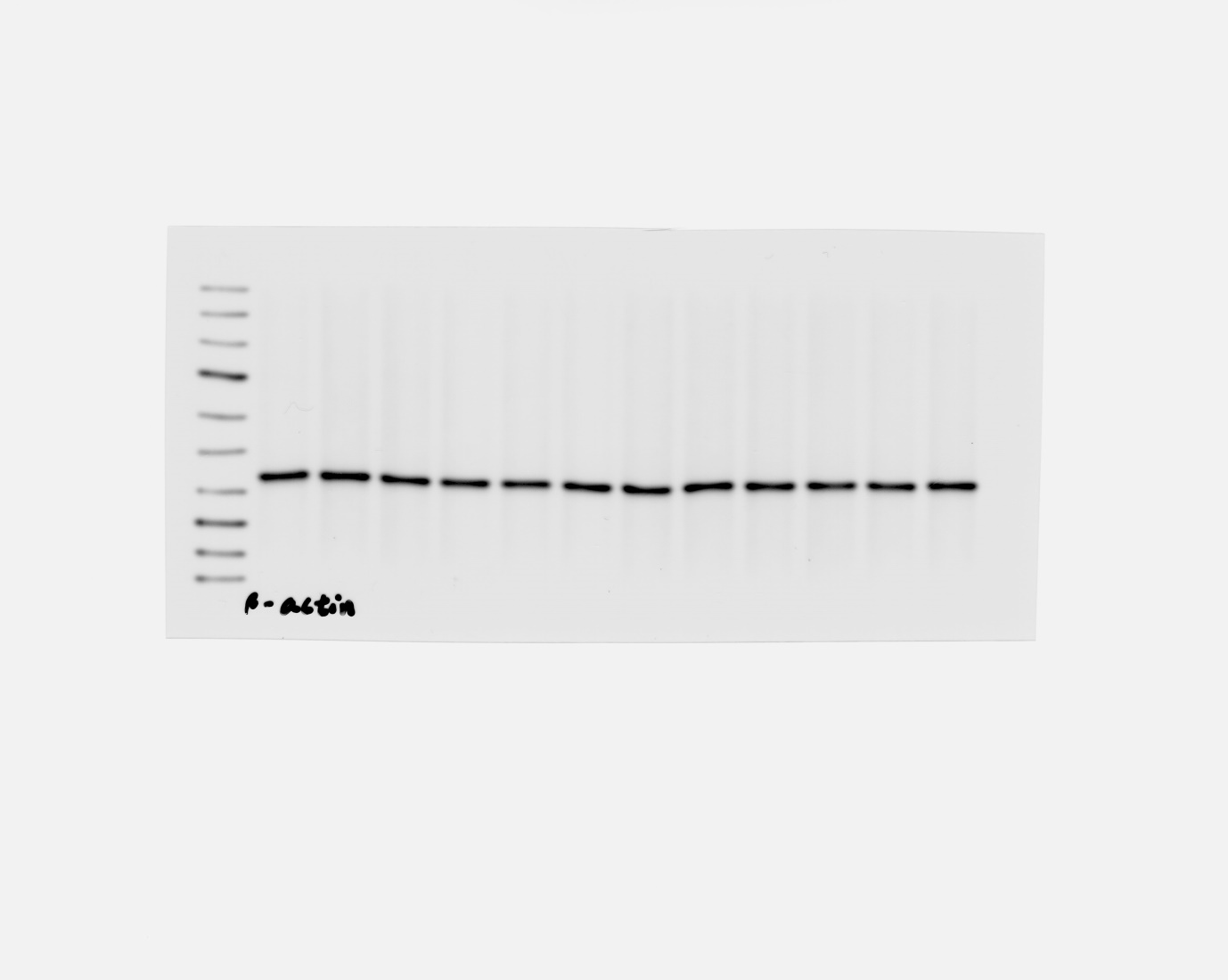

Supplement: Supplementary file 3 — Original Data File [file 41420_2023_1361_MOESM3_ESM.docx]
