# Supplementary material for: Qingre Xingyu recipe exerts inhibiting effects on ulcerative colitis development by inhibiting TNFα/NLRP3/Caspase-1/IL-1β pathway and macrophage M1 polarization
Source: Cell Death Discov. 2023 Mar 8;9:84. doi: 10.1038/s41420-023-01361-w (PMC9995513; doi:10.1038/s41420-023-01361-w)
Supplement: Supplementary file 2 — Supplementary Tables [file 41420_2023_1361_MOESM2_ESM.docx]

**Supplementary Table 1** Information of candidate genes obtained from GeneMANIA

| Symbol | Score | Functions |
| --- | --- | --- |
| NLRP3 | 0.671284 | acute inflammatory response |
| EDN1 | 0.639139 | amide transport |
| CXCL3 | 0.604233 | cell chemotaxis |
| EDN2 | 0.601123 | behavior |
| CCL20 | 0.598724 | cell chemotaxis |
| IL1RN | 0.589977 | acute inflammatory response |
| PLAUR | 0.583093 | negative regulation of multicellular organismal process |
| IER3 | 0.568312 |  |
| CXCL2 | 0.563373 | cell chemotaxis |
| PTX3 | 0.555531 | inflammatory response |
| CCL4 | 0.551666 | behavior |
| IL1B | 0.547164 | acute inflammatory response |
| ICAM1 | 0.515239 | cell activation involved in immune response |

Note: NLRP3, NLR family, pyrin domain containing 3; EDN1, endothelin 1; CXCL3, C-X-C motif chemokine ligand 3; EDN2, endothelin 2; CCL20, C-C motif chemokine ligand 20; IL1RN, interleukin 1 receptor antagonist; PLAUR, plasminogen activator, urokinase receptor; IER3, immediate early response 3; CXCL2, C-X-C motif chemokine ligand; PTX3, pentraxin 3; CCL4, C-C motif chemokine ligand 4; IL-1β, interleukin-1β; ICAM1, intercellular adhesion molecule 1

**Supplementary Table 2** Grouping in microarray GSE53835

| Sample | Type | Treated |
| --- | --- | --- |
| GSM1301820 | Control | Naïve_Comb10_NoDisease_NoTreatment_1 |
| GSM1301821 | Control | Naïve_Comb10_NoDisease_NoTreatment_2 |
| GSM1301822 | Control | Naïve_Comb10_NoDisease_NoTreatment_3 |
| GSM1301823 | Control | Naïve_Comb10_NoDisease_NoTreatment_4 |
| GSM1301824 | Control | Naïve_Comb10_NoDisease_NoTreatment_5 |
| GSM1301825 | Control | Naïve_Comb10_NoDisease_NoTreatment_6 |
| GSM1301826 | Control | Naïve_Comb10_NoDisease_NoTreatment_7 |
| GSM1301827 | Control | Naïve_Comb10_NoDisease_NoTreatment_8 |
| GSM1301828 | Disease | Disease_No_treat_Comb10_1 |
| GSM1301829 | Disease | Disease_No_treat_Comb10_2 |
| GSM1301830 | Disease | Disease_No_treat_Comb10_3 |
| GSM1301831 | Disease | Disease_No_treat_Comb10_4 |
| GSM1301786 | Disease | Disease_No_treat_Comb10_5 |
| GSM1301787 | Disease | Disease_No_treat_Comb10_6 |
| GSM1301788 | Disease | Disease_No_treat_Comb10_7 |
| GSM1301789 | Disease | Disease_No_treat_Comb10_8 |

**Supplementary Table 3** Primer sequence of RT-qPCR

| Genes | Primer sequences |
| --- | --- |
| TNFα (Mus musculus) | F: 5'-CCCTCACACTCAGATCATCTTCT-3' |
|  | R: 5'-GCTACGACGTGGGCTACAG-3' |
| IL-6 (Mus musculus) | F: 5'-TAGTCCTTCCTACCCCAATTTCC-3' |
|  | R: 5'-TTGGTCCTTAGCCACTCCTTC-3' |
| CXCL1 (Mus musculus) | F: 5'-CTGGGATTCACCTCAAGAACATC-3' |
|  | R: 5'-CAGGGTCAAGGCAAGCCTC-3' |
| CXCL12 (Mus musculus) | F: 5'-TTCTTCGAGAGCCACATCGC-3' |
|  | R: 5'-TCAGCCGTGCAACAATCTGA-3' |
| CCL20 (Mus musculus) | F: 5'-AACTGGGTGAAAAGGGCTGT-3' |
|  | R: 5'-GTCCAATTCCATCCCAAAAA-3' |
| CCL5 (Mus musculus) | F: 5'-GCTCCAATCTTGCAGTCGTG-3' |
|  | R: 5'- CAGGACCGAGTGGGAGTAGG -3' |
| ZO-1 (Mus musculus) | F: 5'-ACCCGAAACTGATGCTGTGGATAG-3' |
|  | R: 5'-AAATGGCCGGGCAGAACTTGTGTA-3' |
| IFNγ (Mus musculus) | F: 5'-CCTCAAACTTGGCAATACTCA-3' |
|  | R: 5'-CTCAAGTGGCATAGATGTGGA-3' |
| IL-12 (Mus musculus) | F: 5'-CTCAGAAGCTAACCATCTCCTGG-3' |
|  | R: 5'-CACAGGTGAGGTTCACTGTTTC-3' |
| iNOS (Mus musculus) | F: 5'-CTGCAGCACTTGGATCAGGAACCTG-3' |
|  | R: 5'-GGAGTAGCCTGTGTGCACCTGGAA-3' |
| MCP-1 (Mus musculus) | F: 5'-CCACAACCACCTCAAGCA-3' |
|  | R: 5'-TGAAAGGGAATACCATAACATC-3' |
| Arg1 (Mus musculus) | F: 5'-AAGCCTGGTCTGCTGGAAAAA-3' |
|  | R: 5'-CTGGTTGTCAGGGGAGTGTT-3' |
| IL-10 (Mus musculus) | F: 5'-CGGGAAGACAATAACTGCACCC-3' |
|  | R: 5'-CGGTTAGCAGTATGTTGTCCAGC-3' |
| Claudin-1 (Mus musculus) | F: 5'-GGCTTCTCTGGGATGGATCG-3' |
|  | R: 5'-GCAGCAGTTCACAGGCAAAA-3' |
| Occludin (Mus musculus) | F: 5'-ATGTCCGGCCGATGCTCTC-3' |
|  | R: 5'-TTTGGCTGCTCTTGGGTCTGTAT-3' |
| GAPDH (Mus musculus) | F: 5'-TGGAGAAACCTGCCAAGTATGA-3' |
|  | R: 5'-TGGAAGAATGGGAGTTGCTGT-3' |
| ZO-1 (Homo sapiens) | F: 5'-CGAAGGAGTTGAGCAGGAAA-3' |
|  | R: 5'-ACAGGCTTCAGGAACTTGAG-3' |
| Occludin (Homo sapiens) | F: 5'-GCAAAGTGAATGACAAGCGG-3' |
|  | R: 5'-CACAGGCGAAGTTAATGGAAG-3' |
| Claudin-1 (Homo sapiens) | F: 5'-GGAAGACGATGAGGTGCAGAAG-3' |
|  | R: 5'-GAACGATTCTATTGCCATACC-3' |
| GAPDH (Homo sapiens) | F: 5'-AGAAGGCTGGGGCTCATTTG-3' |
|  | R: 5'-AGGGGCCATCCACAGTCTTC-3' |

Note: TNFα, tumor necrosis factor alpha; IL-6, interleukin 6; CXCL1, C-X-C motif chemokine ligand 1; CXCL12, C-X-C motif chemokine ligand 12; CCL20, C-C motif chemokine ligand 20; CCL5, C-C motif chemokine ligand 5; ZO-1, tight junction protein 1; IFNγ, interferon-gamma; IL-12, interleukin-12; iNOS, inducible nitric oxygen synthase; MCP-1, macrophage cationic peptide 1; Arg1, arginase 1; IL-10, interleukin 10; GAPDH, glyceraldehyde-3-phosphate dehydrogenase; RT-qPCR, reverse transcription-quantitative polymerase chain reaction; F, forward; R, reverse.
